# Ultra-selective uranium separation by in-situ formation of *π*-*f* conjugated 2D uranium-organic framework

**DOI:** 10.1038/s41467-023-44663-4

**Published:** 2024-01-11

**Authors:** Qing Yun Zhang, Lin Juan Zhang, Jian Qiu Zhu, Le Le Gong, Zhe Cheng Huang, Feng Gao, Jian Qiang Wang, Xian Qing Xie, Feng Luo

**Affiliations:** 1 School of Chemistry and Materials Science, East China University of Technology, Nanchang, 330013 China; 2 Key Laboratory of Interfacial Physics and Technology, Shanghai Institute of Applied Physics, Chinese Academy of Sciences, Shanghai, 201800 China; 3 State Key Laboratory of NBC Protection for Civilian, Beijing, 100191 China; 4 National Engineering Research Center for Carbonhydrate Synthesis, Jiangxi Normal University, Nanchang, 330027 China

**Keywords:** Metal-organic frameworks, Polymers, Synthesis and processing

## Abstract

With the rapid development of nuclear energy, problems with uranium supply chain and nuclear waste accumulation have motivated researchers to improve uranium separation methods. Here we show a paradigm for such goal based on the in-situ formation of *π*-*f* conjugated two-dimensional uranium-organic framework. After screening five *π*-conjugated organic ligands, we find that 1,3,5-triformylphloroglucinol would be the best one to construct uranium-organic framework, thus resulting in 100% uranium removal from both high and low concentration with the residual concentration far below the WHO drinking water standard (15 ppb), and 97% uranium capture from natural seawater (3.3 ppb) with a record uptake efficiency of 0.64 mg·g^−1^·d^−1^. We also find that 1,3,5-triformylphloroglucinol can overcome the ion-interference issue such as the presence of massive interference ions or a 21-ions mixed solution. Our finds confirm the superiority of our separation approach over established ones, and will provide a fundamental molecule design for separation upon metal-organic framework chemistry.

## Abstract (web summary)

Methodologies to achieve efficient and selective uranium separation originating from nuclear waste are desirable. Here the authors report the in-situ formation of π-f conjugated 2D uranium-organic framework for uranium separation, leading to superior performance in uranium uptake and outstanding uranium generation efficiency from seawater.

## Introduction

In response to the long-term energy crisis, the development of new energies is now becoming a hot topic. Nuclear energy, because of its high energy density and low carbon pollution, is thus viewed to be one of the effective alternatives^1–5^. However, the sustainable development of nuclear energy is still severely limited by the shortage and insufficient supply chain of uranium. On the other hand, the extensive use of radioactive uranium will also bring serious safety issue, such as environmental pollution and unexpectable diseases^6^. Thereby, it is important to carry out the research of uranium separation from used or new sources^7–13^. Spent fuel, as a typical used source, remains 93.4% unreacted uranium, which thereby can be as a major uranium source through separation, however, such separation was often blocked by the competing adsorption from a broad of metal ions, especially these physically and chemically similar *f*-block ions such as rare earth and other actinide ions^14–16^. Alternatively, seawater reserves abundant uranium, however, the ultralow uranium content down to 3 ~ 4 ppb and the serious vanadium ion interference still prevents the acquisition of uranium from seawater^17–25^. Therefore, significant effort should be devoted to improve uranium separation methods to meet the actual demand.

As an analogue of graphene, 2D (two-dimensional) conjugated metal-organic frameworks (MOFs), are recently receiving increasing attentions, due to its uniqueness in both structure and properties, showing important applications in supercapacitors, batteries, thermoelectric devices, chemiresistive sensors and electrocatalysts^26–28^. The design and synthesis rule of such 2D materials has been realized by the in-plane integration between *π*-conjugated hexa-substituted aromatic cores and late transition metal ions in a square planar coordination geometry^29–31^. Inspired by such way, we can expect the construction of similar 2D *π*-*f* conjugated MOF through a comparable in-plane integration between UO_2_^2+^ ion with a planar coordination in *f* orbitals^32–34^ and proper *π*-conjugated ligands, and further make a hypothesis in uranium separation upon such MOF assembly technology. In this regard, we show herein the molecule design and uranium separation route by means of the concept of in-situ formation of *π*-*f* conjugated uranium-organic framework (UOF).

## Results

### 2D MOF and ligand design

**Fig. 1:**
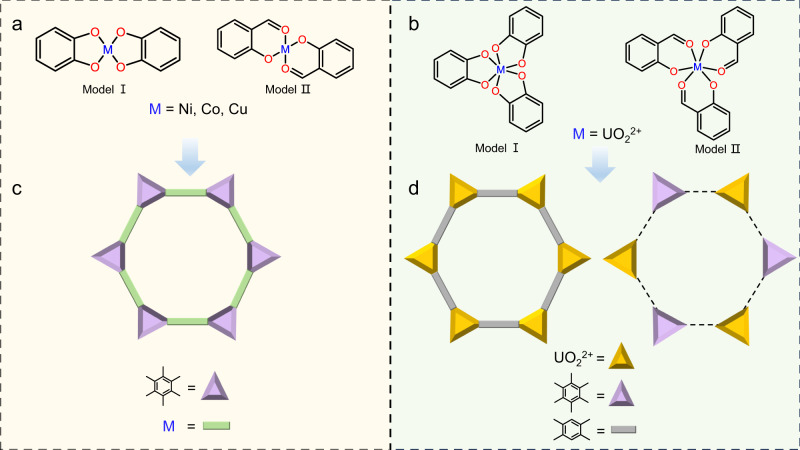
Molecular design of conjugated 2D MOFs. **a** *π*-*d* in-plane integration in the manner of model I and II. **b** View of the *π*-*d* conjugated 2D MOFs by means of [3 + 2] coupling with *π*-conjugated organic ligand as three-connected node and metal ions in the square planar coordination geometry as two-connected linker. **c** *π*-*f* in-plane integration in the manner of model I and II. **d** View of the *π*-*f* conjugated 2D MOFs by means of [3 + 2] or [3 + 3] coupling with UO_2_^2+^ ion as three-connected node and *π*-conjugated organic ligand as two-connected linker or three-connected node.

**Fig. 2:**
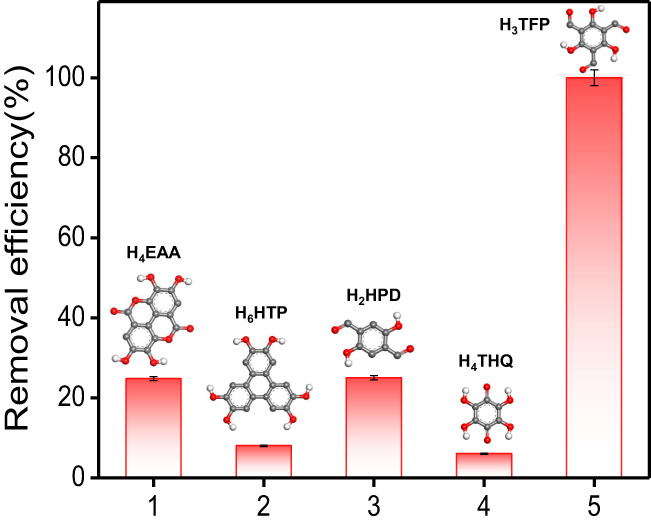
Ligands used in this work. A screen of various organic ligands for UO_2_^2+^ capture upon in-situ formation of MOF method. The error bars indicate the standard deviation (*n* = 3).

Previous research has revealed the molecular design rule for conjugated 2D MOF^29–31^. The key was the in-plane coupling between metal ions and organic ligands in a defined and periodic manner. It was found that these metal ions such as Ni^2+^, Co^2+^, and Cu^2+^ in the square planar coordination geometry and these hexa-substituted planar conjugated benzenes such as 1,3,5-triformylphloroglucinol (H_3_TFP) and 2,3,6,7,10,11-hexahydroxytriphenylene (H_6_HTP) meet the in-plane [3 + 2] coupling, where the organic ligands take the chelate coordination mode (model I and II, Fig. 1a) and act as three-connecting nodes, while metal ions act as two-connecting linker, finally resulting in the *π*-*d* conjugated 2D MOF (Fig. 1b)^35,36^. Different from these transition metal ions of Ni^2+^, Co^2+^, and Cu^2+^ that use *d* orbit for coordination, UO_2_^2+^ ion, the common uranium type, affords the planar coordination feature in the *f* orbit and theoretically conducts more orbit to participate in coordination, generally showing the six-coordination fashion. Corresponding to this is the distinct coordination mode (model I and model II, Fig. 1c) between organic ligands and UO_2_^2+^ ions and 2D MOFs generated by [3 + 2] or [3 + 3] coupling (Fig. 1d), where UO_2_^2+^ ions act as three-connecting node and organic ligands act as two-connecting linker or three-connecting nodes, respectively. In this regard, we screened five comparable, planar conjugated ligands, composed of hexa-substituted H_3_TFP, H_6_HTP, and H_3_THQ (tetrahydroxyquinone), tetra-substituted H_2_HPD (2,5-dihydroxyterephthalaldehyde) and H_4_EAA (ellagic acid). As shown in Fig. 2, all these substrates were found to be effective in UO_2_^2+^ capture from a 100 ppm UO_2_^2+^ solution after contacting for 24 h, giving a hierarchy of H_3_TFP (100%) > H_2_HPD (25.6%) > H_4_EAA (24.8%) > H_6_HTP (8.3%) > H_3_THQ (6%), implying H_3_TFP being the best one. Accordingly, the next investigation is just focused on H_3_TFP ligand.

To clarify the difference in UO_2_^2+^ capture for these organic ligands, we then carried out the calculation on the binding energy (ΔG) of these organic ligands with UO_2_^2+^ ion by density functional theory (DFT) method. A planar six-coordination model of one UO_2_^2+^ ion coordinated by three these organic ligands was used to carried out DFT calculation. The optimized coordination structures of them were shown in Supplementary Fig. 1. The binding energy (*ΔG*) gives a hierarchy of H_4_THQ (−0.29 eV)>H_6_HTP (−0.69 eV)>H_4_EAA (−1.02 eV)>H_2_HPD (−2.99 eV)>H_3_TFP (−3.91 eV). Generally, negative binding energy (*ΔG*) suggests the reaction thermodynamically spontaneous, while this also obeys a rule, *viz*. the more negative, and the stronger the binding. Thus, the negative *ΔG* values mean that all these organic ligands can capture UO_2_^2+^ ion through coordination, which is consistent with the experimental results, while the smallest and biggest *ΔG* value in, respectively, H_3_TFP and H_4_THQ means the strongest and weakest binding and consequently the biggest and smallest UO_2_^2+^ uptake, which is also in good agreement with the experimental results. Moreover, the hierarchy in binding energy (*ΔG*) is also in accord with the hierarchy in the UO_2_^2+^ uptake, confirming the adsorption of UO_2_^2+^ ions by these organic ligand obeying the defined planar coordination principle. In addition, seen from the optimized coordination structures of them, it is found that the chelate coordination from the combination of one aldehyde oxygen and one hydroxyl oxygen (such as H_4_TFP and H_2_HPD) is more beneficial for strengthening U-O coordination and planar coordination configuration over the chelate coordination from two hydroxyl oxygens (such as H_4_EAA, H_6_HTP, H_4_THQ), due to steric hindrance effect. And this could be the key to determine the UO_2_^2+^ uptake performance. Moreover, the bigger conjugated organic molecule is beneficial for strengthening planar coordination configuration and consequently enhancing UO_2_^2+^ uptake, e.g., H_6_HTP and H_4_EAA vs. H_4_THQ.

### Adsorption kinetics

**Fig. 3:**
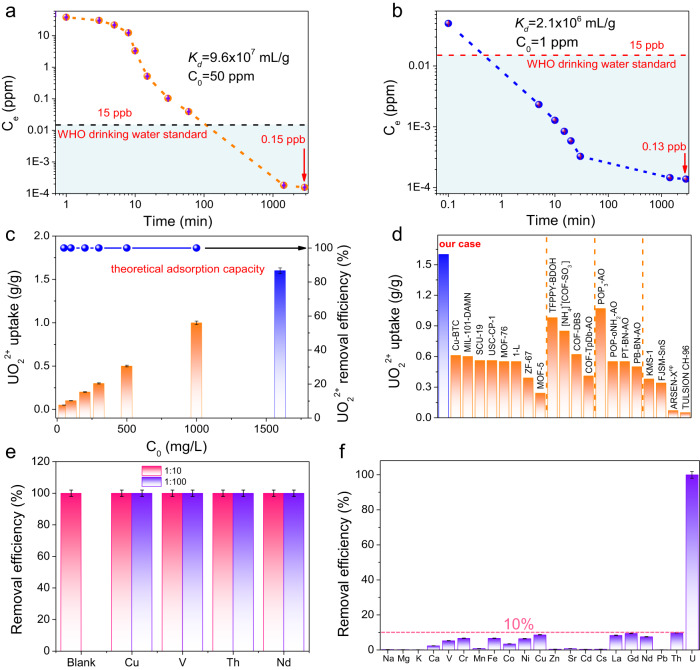
Uranium adsorption upon H_3_TFP. **a** Adsorption kinetics from 50 ppm U solution upon H_3_TFP adsorbent. **b** Adsorption kinetics from 1 ppm U solution upon H_3_TFP adsorbent. **c** Adsorption capacity of H_3_TFP adsorbent. Highlight in blue represents the theoretical adsorption capacity. **d** A comparison in U uptake capacity between reported U adsorbents and our case. **e** Influence of interfering ions on U adsorption. **f** Selective U capture from a 21-ions mixture solution. The error bars indicate the standard deviation (*n* = 3).

Fast adsorption process is usually the way we want. We then tested the adsorption kinetics of H_3_TFP ligand from a 50 ppm U(VI) solution with pH = 3 through using 10 mg adsorbent (Fig. 3a). Notably, the adsorption equilibrium was finished within 15 min with 100% removal efficiency, suggesting ultrafast adsorption kinetics, due to the thermodynamicly spontaneous chemical reaction through coordination assembly between UO_2_^2+^ ions and H_3_TFP ligand. After extending the contact time to 24 h and 48 h, we found that the residual uranium concentration was decreased down to ultralow level of 0.18 ppb and 0.15 ppb, respectively, far lower than the World Health Organization (WHO) standard (15 ppb) for uranium content in drinking water. In light of this data, the distribution coefficient, *K*_*d*_, was calculated up to 9.6 × 10^7 ^mL/g (a *K*_*d*_ value exceeding 1.0 × 10^5 ^mL/g is usually considered as excellent adsorbent), implying strong affinity between adsorbents and uranium resulted from the strong U-O coordination interactions. This value ranks the top level among all established uranium adsorbents, including POP_1_-AO (1.1 × 10^6^ mL/g)^37^, MIGPAF-13 (2.0 × 10^6^ mL/g)^38^, SMON-PAO (3.7 × 10^5^ mL/g)^39^, and PIDO/NF (2.8 × 10^5^ mL/g)^7^. This exceptional uranium removal ability was further attested for a low concentration of U(VI) solution (1 ppm), giving a residual uranium concentration of 2.3 ppb after 5 min and 0.13 ppb after 48 h (Fig. 3b), also far exceeding the WHO standard. Big *K*_*d*_ value up to 2.1 × 10^6^ mL/g was also observed.

### Adsorption capacity

Large adsorption capacity is also one of the goals pursued by adsorbent. Next, we investigated the adsorption capacity upon H_3_TFP adsorbent from a uranium solution with concentration of 10–1000 ppm. Interstingly, this adsorbent enabled 100% removal for all these uranium solutions (Fig. 3c), which is never observed in the literature. The experimental uranium uptake capacity was as high as 1.0 g/g. If taking the formation of UOF through [3 + 3] coupling into account, the theoretical adsorption capacity is estimated as big as 1.6 g/g, which surpasses all established uranium adsorbents (Fig. 3d, Supplementary Table 1)^40^, including these benchmark adsorbents such as MIL-101-DAMN (0.60 g/g)^41^, Cu-BTC (0.61 g/g)^42^, TFPPy-BDOH (0.98 g/g)^43^, and POP_3_-AO (1.07 g/g)^37^. This also confirmed the advantage of our method over established approaches.

### pH effect

It was found that pH value showed significant effect on the uranium uptake performance (Supplementary Fig. 2). The optimal performance was found under pH = 3 and 5, whereas both increasing acidity and alkalinity would lead to sharp decrease in the uranium uptake, for example, 100% uranium removal under pH = 3 and 5 *vs*. 12.6% uranium removal under pH = 1 or 21.8% uranium removal under pH = 9. High acidity leading to a sharp decrease in uranium uptake is mainly due to the protonation that will significantly affect the coordination of H_3_TFP hydroxyl groups, and high alkalinity resulting in a sharp decrease in uranium uptake is mainly due to the solution of H_3_TFP under such condition that will significantly affect the UOF formation.

### Selectivity towards UO_2_^2+^

As we know, divalent copper and trivalent iron ion were found to construct 2D MOF with H_3_TFP ligands^35^, while the presence of trivalent 4f ions, or tetravalent thorium ion^23^, or vanadium ion were often found to give significant effect on UO_2_^2+^ capture^37,38^. Thereafter, it is important to research the uranium adsorption performance in the presence of these interfering ions. Then, we carried out the uranium adsorption experiments form a 10 ppm uranium solution under the presence of massive other interfering ions with U/M (M = Cu^2+^, Fe^3+^, Nd^3+^, Th^4+^, and VO_3_^−^) ratio from 1:10 to 1:100 (Fig. 1e). Notably, no decrease in the uranium uptake was observed in the presence of other interfering ions, even expanding the U/M ratio to 1:100, completely excluding the influence of interfering ions. Such selectivity towards UO_2_^2+^ ion over other ions is highly rare in the literature^44,45^. Furthermore, we carried out a selectivity test from a 21-ions mixture solution, including in Na^+^, K^+^, Cs^+^, Mg^2+^, Ca^2+^, Sr^2+^, Zn^2+^, Cd^2+^, Pd^2+^, Mn^2+^, Cu^2+^, Co^2+^, Ni^2+^, Cr^3+^, Fe^3+^, La^3+^, Gd^3+^, Nd^3+^, Th^4+^, UO_2_^2+^, and VO_3_^-^ (Fig. 1f). It was found that UO_2_^2+^ was 100% captured, whereas other ions just gave less than 10% removal efficiency, suggesting highly selective capture of UO_2_^2+^ ion over other 20 ions.

The selectivity of UO_2_^2+^ over Fe^3+^, Nd^3+^, Th^4+^, and VO_3_^−^ can be easy to understand, since UO_2_^2+^ is planarly coordinated and can effectively construct the π-f conjugated 2D uranium-organic framework, whereas other ions such as Fe^3+^, Nd^3+^, Th^4+^, and VO_3_^−^ are spherical coordinated and can not construct corresponding π-d/f conjugated 2D metal-organic framework. However, Cu^2+^ ions own the planar coordination feature, and can form the π-d conjugated 2D metal-organic framework, which will theoretically result in considerably competitive adsorption with UO_2_^2+^ ion. Thereby, to understand the UO_2_^2+^/Cu^2+^ selectivity in this work, DFT calculation on binding energy was carried out (Supplementary Fig. 3). The negative binding energy (*ΔG*) of −1.16 eV implies the adsorption potential of H_3_TFP ligand towards Cu^2+^ ions. But the binding towards Cu^2+^ ion is far weaker than that towards UO_2_^2+^ ion, as evidenced by the *ΔG* value (−1.16 eV) in Cu^2+^ ion that is far bigger than that (−3.91 eV) in UO_2_^2+^ ion; thus H_3_TFP ligand can enable selective UO_2_^2+^ capture over Cu^2+^ ion.

### Recycle use and liquid-liquid extraction

**Fig. 4:**
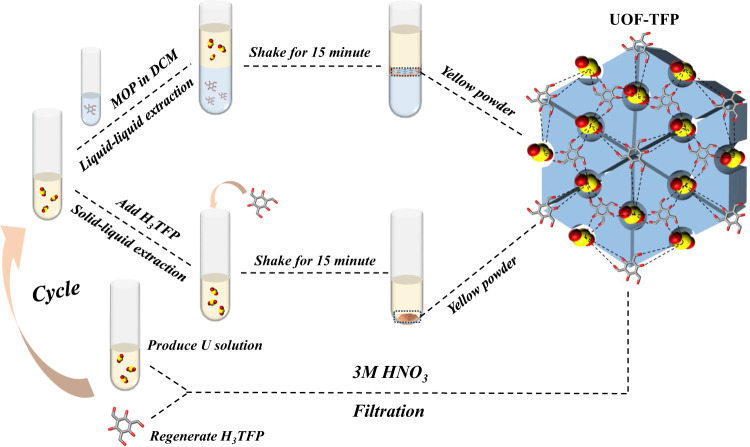
Our MOF routes. View of the adsorption-desorption cycle by means of our separation approach in both solid- and liquid-extraction routes.

More interestingly, H_3_TFP adsorbent can be conveniently recovered in the form of precipitate through using 3 M HNO_3_ as eluent, while the adsorbed uranium on adsorbent can be 100% desorbed into solution in the form of UO_2_(NO_3_)_2_, possibly due to a disassembly of UOF with the breakage of all U-O coordination bonds. Such complete solid-liquid separation (Fig. 4) facilitates our following recycle use to an ideal form, as evidenced by observation of no decrease in both uranium adsorption and adsorbent recovery from a 100 ppm uranium solution after repeating adsorption-desorption cycles (Fig. 4) for 11 times (Supplementary Fig. 4).

Interestingly, although H_3_TFP is insoluble in water, however it gave good solubility in many organic solvents, involved in dichloromethane (DCM), toluene (PhMe), ortho-dichlorobenzene (ODCB), p-xylene (PX), m-xylene (MX), ortho-xylene (OX), and trimethylbenzene (TB). Thus, we further developed the liquid-extraction route (Fig. 4) and the results were shown in Supplementary Fig. 5. Notably, such liquid-extraction route was found to be also effective for uranium capture. Especially, H_3_TFP in DCM and ODCB was found to give 100% removal for a 50 ppm uranium solution within 15 min. This result is comparable with that observed in the solid-extraction route. We also extended the contacting time up to 48 h for H_3_TFP in DCM, and found low residual concentration of uranium (1.24 ppb), which is also far below the WHO standard of drinking water.

In both solid- and liquid-extraction routes, a key step in the desorption process is the solid-liquid separation between UO_2_(NO_3_)_2_ solution and H_3_TFP precipitation regeneration from desorption. Thus, it is important to reveal the solubility of H_3_TFP in water. As shown in Supplementary Fig. 6, it was clear that H_3_TFP is completely soluble in ODCM, but insoluble in water. This can be further attested by UV-visiblespectral test (Supplementary Fig. 7), where H_3_TFP in ODCM gave a strong adsorption peak at 320 nm, while no obvious adsorption was observed for H_3_TFP in water.

### Extraction of uranium from seawater

To confirm the practical application of our uranium separation method, we further explored uranium capture performance from natural seawater. It was found that the use of 10 mg H_3_TFP adsorbent can reduce a 10 L natural seawater (3.3 ppb U) to 0.1 ppb after contacting for 5 days, showing uranium uptake as high as 3.2 mg/g. If considering the time cost, the uranium uptake efficiency is 0.64 mg·g^−1^·d^−1^. Such value far exceeds the top adsorbents (Supplementary Table 2) such as PPH-OP (0.36 mg·g^−1^·d^−1^)^46^, AP-PIM-1 (0.32 mg·g^−1^·d^−1^)^47,48^, MIGPAF-13 (0.28 mg·g^−1^·d^−1^)^38^, and POP_1_-AO (0.15 mg·g^−1^·d^−1^)^37^. In addition, we also conducted the uranium extraction under a longer contacting time from a 20 L natural seawater (3.3 ppb U) using 10 mg H_3_TFP adsorbent. Impressively, 97% uranium can be captured after 10 days, giving uranium uptake as high as 6.4 mg/g, while extending contacting time up to 14 days did not increase uranium uptake. A comparison in uranium uptake between established materials and our case is shown in Supplementary Table 3, which clearly suggests our case with the location of top level in the field of uranium extraction from seawater. Moreover, we should also consider the economy of uranium extraction from seawater. In generally, the manufacturing cost of adsorbents dominates the total cost for uranium extraction from seawater. For our case, the cost of preparing H_3_TFP adsorbent is as low as 1.3 $/g, confirming its economic feasibility. Such value is far below the current benchmark adsorbents such as COF 4 P (4.7 $/g)^14^ and COF-4 (2.7 $/g)^15^.

## Discussion

### Uranium capture mechanism

As mentioned above, the uranium capture upon H_3_TFP adsorbent majorly obeys the rule of in-situ formation of *π*-*f* conjugated 2D UOF, where the key is the assembly between *π*-conjugated H_3_TFP ligands and *f* ions of UO_2_^2+^ in the defined and periodic manner. The use of *f* orbit to participate in bonding for UO_2_^2+^ ions suggests the formation of considerably more ionic compounds than transition metals, meaning that UO_2_^2+^ ions are more likely to form crystalline MOFs than transition metals;^26,47^ this can reasonably explain the selectivity of uranium over transition metal ions such as Cu^2+^ and Fe^3+^, even though these metal ions can also form *π*-*d* conjugated 2D MOFs. The *f* orbit in UO_2_^2+^ ion shows the in-plane coordination feature, meeting the in-plane assembly, whereas other *f* ions such as rare-earth (trivalent state) and thorium (tetravalent state) ions afford sphere coordination feature^49,50^, which fully excludes the in-plane assembly; this can reasonably explain the selectivity of uranium over Nd^3+^ and Th^4+^ ions. Similarly, vanadium ions also own the sphere coordination feature, thus excluding the in-plane assembly and finally leading to the big selectivity of UO_2_^2+^ over VO_3_^−^ ion.

To confirm the formation of *π*-*f* conjugated 2D UOF during the uranium adsorption upon H_3_FTP adsorbent, we carried out a series of characterizations on the uranium-loaded samples (namely UOF-TFP), including in IR (infrared spectrum), XPS (X-ray photoelectron spectroscopy), PXRD (powder X-ray diffraction), TG (thermogravimetric analysis), N_2_ adsorption, SEM-EDS (scanning electron microscope *plus* energy-dispersive X-ray spectroscopy), and TEM (transmission electron microscope). IR spectra disclosed new peak at 922 cm^−1^, belonging to the antisymmetric vibration of uranyl ions, which, relative to UO_2_(NO_3_)_2_·6H_2_O with peak at 960 cm^−1^ (Supplementary Fig. 8)^44,45^, shows big red shift, implying strong coordination interactions between H_3_TFP adsorbent and uranyl ions. And coordination with the participation of both aldehyde and phenolic groups of H_3_TFP adsorbent can be further read out from IR peaks showing big shift or almost disappearance for aldehyde and phenolic groups, relative to free H_3_TFP molecule. The success in uranium capture upon H_3_TFP adsorbent can be further reflected in XPS spectrum, showing typical UO_2_^2+^ peaks at 381.8 eV and 392.7 eV for *U4f*_*7/2*_ and *U4f*_*5/2*_, respectively (Supplementary Fig. 9). The values are significantly lower than that of UO_2_(NO_3_)_2_·6H_2_O (382.5 eV and 393.4 eV)^46,47^, also confirming the strong coordination interactions between H_3_TFP ligands and UO_2_^2+^ ions. The presence of two satellite peaks at higher binding energy, relative to *U4f*_*7/2*_ and *U4f*_*5/2*_, confirms the U(VI) oxidation state in UOF-TFP.

**Fig. 5:**
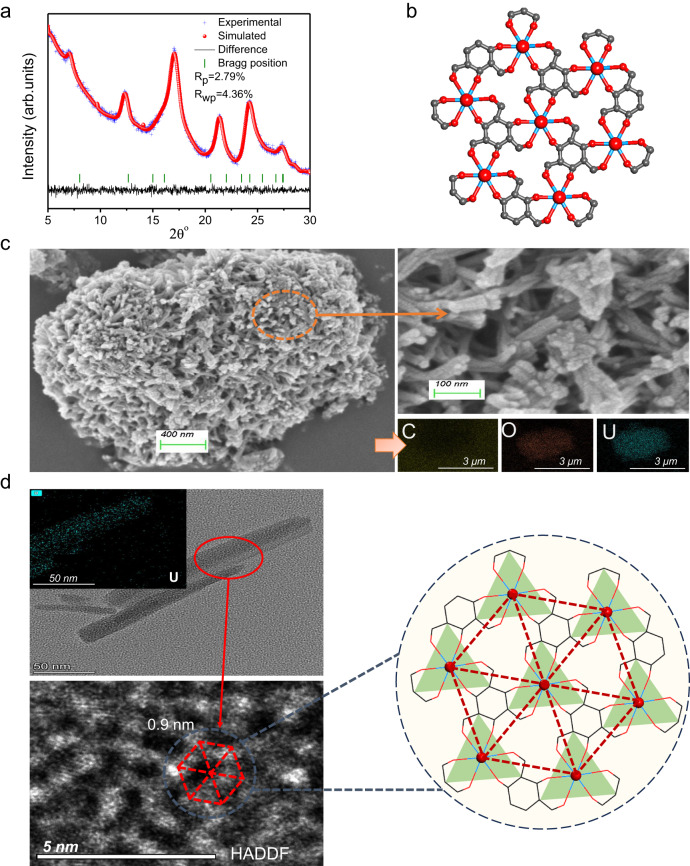
Characterizations of UOF-TFP. **a** Experimental PXRD patterns and pawley refinements. **b** View of *π*-*f* conjugated 2D UOF-TFP. **c** SEM images and element mapping of UOF-TFP. **d** STEM-HADDF images and U element mapping of UOF-TFP. The highlight is the 2D hexagonal lattice of uranium ions.

The single crystal of UOF-TFP can not be obtained, blocking us to access its exact structure. But, we can simulate its structure from powder X-ray diffraction (PXRD) *plus* pawley refinements^44,45^. PXRD pattern showed high crystallinity of UOF-TFP. In light of the coordination feature of both UO_2_^2+^ ions and H_3_TFP ligands, we then proposed a 2D MOF model, and then Pawley refinements were carried out on the experimental PXRD (Fig. 5a). It was found that the refined PXRD pattern was in good agreement with the experimental counterpart, as evidenced by the small difference and reasonable *R*_*p*_ = 2.65% and *R*_*wp*_ = 3.78% values. The layered nets in UOF-TFP showed a staggered orientation (AB stacking). The structure of UOF-TFP was shown in Fig. 5b. UO_2_^2+^ ions afforded the planar six-coordination by three TFP^3−^ ligands using three phenolic oxygen and three aldehyde oxygen atoms. The TFP^3-^ ligands afforded a chelate coordination mode through using adjacent one phenolic oxygen and one aldehyde oxygen atom to fix one UO_2_^2+^ ion. The U-O bond length of 2.40–2.70 Å is in the normal range^33,34^. The *π*-*f* conjugated 2D net was built in a [3 + 3] way that each TFP^3−^ ligand connects to three UO_2_^2+^ ions, while each UO_2_^2+^ ion also connects to three TFP^3−^ ligands, resulting in an overall binodal *hcb* net. Moreover, such AB stacking led to small void among layers (Supplementary Fig. 10), occupied by water molecules. TG test disclosed the loss of solvent water molecules before 230 °C, and decomposition of UOF-TFP occurred after 310 °C (Supplementary Fig. 11). In addition, the micoporosity was confirmed by N_2_ adsorption at 77 K (Supplementary Fig. 12).

More impressively, such *π*-*f* conjugated 2D net can be intuitively read out by STEM-HADDF test. First, SEM disclosed the nanorod morphology of UOF-TFP (Fig. 5c). EDS element analysis clearly disclosed the presence of uranium element, and the U/C atom ratio is well consistent with the structural data, confirming the accuracy of our structural simulation. Furthermore, such nanorod morphology can be also clearly read out from TEM images. Impressively, UO_2_^2+^ ions and its location can be clearly read out from STEM-HADDF images (Fig. 5d), where the UO_2_^2+^ ions are approximately arranged in a *hcb* fashion with a distance of ca. 0.9 nm between two adjacent U ions, which is similar to that observed in the simulated structure data with a distance of ca. 0.81 nm.

**Fig. 6:**
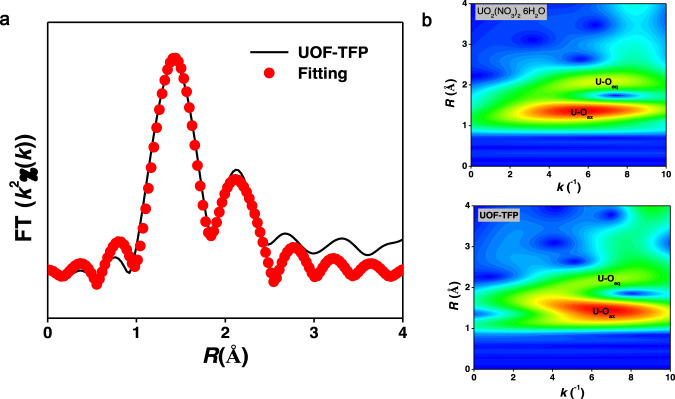
EXZAF of UOF-TFP. **a** Experimental EXZAF data (red) and fitting results (black). **b** View of coordination surrounding of UOF-TFP and UO_2_(NO_3_)_2_·6H_2_O, where U-O_ax_ represents the U = O coordination bonds, U-O_eq_ represents the U-O_nitrate_ *plus* U-O_water_ in UO_2_(NO_3_)_2_·6H_2_O and U-O_hydroxyl_ *plus* U-O_aldehyde_ in UOF-TFP.

In addition, to gain deep insight into the local coordination sphere of the uranium species, U K-edge X-ray absorption near-edge structure (XANES, Supplementary Fig. 13) and extended X-ray absorption fine structure (EXAFS, Supplementary Fig. 14) spectroscopy were performed. UO_2_(NO_3_)_2_·6H_2_O was used as the reference standard. It was found that UOF-TFP rendered comparable XANES and EXAFS spectra with that observed in UO_2_(NO_3_)_2_·6H_2_O, confirming their similarity in the valence state and coordination surrounding of uranium. As we know, uranium in UO_2_(NO_3_)_2_·6H_2_O is hexavalent in the form of UO_2_^2+^, and UO_2_^2+^ in UO_2_(NO_3_)_2_·6H_2_O takes planar six coordination with two NO_3_^-^ ions and two coordination water molecules, where each NO_3_^−^ ion displays a chelate coordination mode with two NO_3_^−^ oxygen atoms to fix one UO_2_^2+^ ion. Accordingly, we can deduce UO_2_^2+^ ions with the planar six coordination in UOF-TFP (Supplementary Table 4). High-quality fits of the EXAFS data for UOF-TFP (Fig. 6a, b) strongly suggest an eight coordination of uranium, where two U-O coordinations with bond length of 1.85 ± 0.01 Å are assigned to U = O bond of UO_2_^2+^ ion, other six U-O coordinations with longer bond length are assigned to the planar U-O coordination from TFP^3-^ ligand. This also reveals two types of U-O bond with equal numbers in the bond length of 2.42 ± 0.02 Å and 2.51 ± 0.01 Å, respectively. We then comparied the results from EXAFS data with the results from structural simulation, and found that the U = O bond of UO_2_^2+^ ion form EXAFS data is well consistent with the results from structural simulation (1.84 Å), while the bond length of 2.42 ± 0.02 Å and 2.51 ± 0.01 Å from EXAFS data is close to U-O_hydroxyl_ bond (2.40 Å, hydroxyl oxygen of TFP^3−^ as coordination atom) and U-O_aldehyde_ bond (2.70 Å, aldehyde oxygen of TFP^3−^ as coordination atom) from structural simulation. Thereby, the structure of UOF-TFP can be once again confirmed by EXAFS results.

In summary, we have demonstrated a proof-of-concept of in-situ formation of *π*-*f* conjugated 2D UOF for targeting at highly efficient and selective uranium separation. Taking both coordination and self-assembly chemistry into account, through using the simple but powerful organic ligand of H_3_TFP molecule, we can easily appoint the self-assembly between *π*-conjugated H_3_TFP ligands and in-plane coordinated *f* UO_2_^2+^ ions in a defined and periodic manner, leading to the rapid and massive formation of UOF. Such feature finally offered an ideal uranium separation process, as evidenced by its superior performance, including in recorded theoretical uptake capacity, ultrafast adsorption kinetics, large distribution coefficient, big selectivity, and long-term repeatability. This work not only outlines a concept of how to use MOF chemistry to solve practical problems, but also points out a promising direction in the field of uranium separation.

## Methods

### Materials and measurements

H_3_TFP (99%), H_2_HPD (99%) and H_6_HTP (99%) were purchased from Jilin Chinese Academy of Sciences - Yanshen Technology Co., Ltd. H_4_THQ (99%), H_4_EAA (99%), UO_2_(NO_3_)_2_·6H_2_O (99%), and these organic solvents (99%) were purchased from Aladdin Biochemical Technology Co., Ltd. These were used as received without further purification. X-ray powder diffraction were collected by a Bruker AXSD8 Discover powder diffractometer at 40 kV, 40 mA for Cu Kλ (λ = 1.5406 Å). The simulated powder patterns were calculated by Mercury 1.4. Infrared Spectra (IR) were measured by a Bruker VERTEX70 spectrometer in the 700–3600 cm^−1^ region. The gas adsorption isotherms were collected on a Belsorp-max. Ultrahigh-purity-grade (>99.999%) N_2_ gases were used during the adsorption measurement. The analyses of concentrations of U ions in the solution was carried out by ThermoFisher iCap7600 ICP-OES or iCap RQplus ICP-MS instruments. X-ray photoelectron spectra (XPS) were collected by Thermo Scientific ESCALAB 250 Xi spectrometer. Scanning electron microscopy (SEM) images were recorded on a Hitachi SU 8100 Scanning Electron Microscope. Transmission Electron Microscope (TEM) was recorded on a Talos F200x from Thermo Fisher Scientific. UV-vis spectroscopy were recorded at room temperature on a SHIMADZU UV-2700 spectrophotometer.

### Supplementary information

Supplementary Information

Peer review file

### Source data

Source data

## Data Availability

The authors declare that all the data supporting the findings of this study are available within the article (and Supplementary Information Files), or available from the corresponding author on request. All the data generated in this study have been deposited in the Figshare database under [10.6084/m9.figshare.24085956]. Source data are provided with this paper.

## References

[1] Hoffert MI (1998). Energy implications of future stabilization of atmospheric CO_2_ content. Nature.

[2] DeCanio SJ, Fremstad A (2011). Economic feasibility of the path to zero net carbon emissions. Energy Policy.

[3] Nuclear key to a clean energy future: IEA World Energy Outlook (2016). https://www.iea.org/reports/world-energy-outlook-2016.

[4] Nifenecker H (2011). Future electricity production methods. Part 1: Nuclear energy. Rep. Prog. Phys..

[5] Sovacool BK (2008). Valuing the greenhouse gas emissions from nuclear power: a critical survey. Energy Policy.

[6] Craft E (2004). Depleted and natural uranium: chemistry and toxicological effects. J. Toxicol. Environ. Health B Crit. Rev..

[7] Wang D (2018). Significantly enhanced uranium extraction from seawater with mass produced fully amidoximated nanofiber adsorbent. Adv. Energy Mater..

[8] Sun Q (2018). Covalent organic frameworks as a decorating platform for utilization and affinity enhancement of chelating sites for radionuclide sequestration. Adv. Mater..

[9] Sun Q (2018). Bio-inspired nano-traps for uranium extraction from seawater and recovery from nuclear waste. Nat. Commun..

[10] Wang XX (2019). Synthesis of novel nanomaterials and their application in efficient removal of radionuclides. Sci. China Chem..

[11] Yuan Y (2018). Molecularly imprinted porous aromatic frameworks and their composite components for selective extraction of uranium ions. Adv. Mater..

[12] Li J (2018). Metal-organic framework-based materials: superior adsorbents for the capture of toxic and radioactive metal ions. Chem. Soc. Rev..

[13] Zheng T (2017). Overcoming the crystallization and designability issues in the ultrastable zirconium phosphonate framework system. Nat. Commun..

[14] Yang H (2023). Tuning local charge distribution in multicomponent covalent organic frameworks for dramatically enhanced photocatalytic uranium extraction. Angew. Chem. Int. Ed..

[15] Chen ZS (2023). Tuning excited state electronic structure and charge transport in covalent organic frameworks for enhanced photocatalytic performance. Nat. Commun..

[16] Zhang HL (2019). Three mechanisms in one material: uranium capture by a polyoxometalate-organic framework through combined complexation, chemical reduction, and photocatalytic reduction. Angew. Chem. Int Ed..

[17] Wang ZY (2020). Constructing an ion pathway for uranium extraction from seawater. Chem.

[18] Feng LJ (2022). In situ synthesis of uranyl-imprinted nanocage for selective uranium recovery from seawater. Angew. Chem. Int. Ed..

[19] Cui WR (2020). Regenerable covalent organic frameworks for photo-enhanced uranium adsorption from seawater. Angew. Chem. Int. Ed..

[20] Yuan Y (2019). A molecular coordination template strategy for designing selective porous aromatic framework materials for uranyl capture. ACS Cent. Sci..

[21] Yuan Y, Zhu GS (2019). Porous aromatic frameworks as a platform for multifunctional applications. ACS Cent. Sci..

[22] Kalaj M (2020). MOF-polymer hybrid materials: from simple composites to tailored architectures. Chem. Rev..

[23] Yuan YH (2020). A Bio-inspired nano-pocket spatial structure for targeting uranyl capture. Angew. Chem. Int. Ed..

[24] Chen Z (2018). N, P, and S codoped graphene-like carbon nanosheets for ultrafast uranium (VI) capture with high capacity. Adv. Sci..

[25] Yu QH (2019). A universally applicable strategy for construction of anti-biofouling adsorbents for enhanced uranium recovery from seawater. Adv. Sci..

[26] Skorupskii G (2020). Efficient and tunable one-dimensional charge transport in layered lanthanide metal-organic frameworks. Nat. Chem..

[27] Sheberla D (2016). Conductive MOF electrodes for stable supercapacitors with high areal capacitance. Nat. Mater..

[28] Feng DW (2018). Robust and conductive two-dimensional metal-organic frameworks with exceptionally high volumetric and areal capacitance. Nat. Energy.

[29] Dou JH (2017). Signature of metallic behavior in the metal-organic frameworks M_3_(hexaiminobenzene)_2_ (M = Ni, Cu). J. Am. Chem. Soc..

[30] Sheng SC (2022). A one-dimensional conductive metal-organic framework with extended π-d conjugated nanoribbon layers. Nat. Commun..

[31] Xing GL (2023). Conjugated nonplanar copper-catecholate conductive metal-organic frameworks *via* contorted hexabenzocoronene ligands for electrical conduction. J. Am. Chem. Soc..

[32] Lv K, Fichter S, Gu M, März J, Schmidt M (2021). An updated status and trends in actinide metal-organic frameworks (An-MOFs): From synthesis to application. *Coor*. Chem. Rev..

[33] Mei S (2023). Assembling a heterobimetallic actinide metal-organic framework by a reaction-induced preorganization strategy. Angew. Chem. Int. Ed..

[34] Hanna SL (2022). Discovery of spontaneous de-interpenetration through charged point-point repulsions. Chem.

[35] Zhang Z (2022). Ultrafast interfacial self-assembly toward supramolecular metal-organic films for water desalination. Adv. Sci..

[36] Xie LS, Skorupskii G, Dincă M (2020). Electrically conductive metal-organic frameworks. Chem. Rev..

[37] Song YP (2021). Nanospace decoration with uranyl-specific “hooks” for selective uranium extraction from seawater with ultrahigh enrichment index. ACS Cent. Sci..

[38] Wang ZY (2021). Constructing uranyl-specific nanofluidic channels for unipolar ionic transport to realize ultrafast uranium extraction. J. Am. Chem. Soc..

[39] Yuan YH (2019). Charge balanced anti-adhesive polyacrylamidoxime hydrogel membrane for enhancing uranium extraction from seawater. Adv. Funct. Mater..

[40] Mei DC, Liu LJ, Yan B (2023). Adsorption of uranium (VI) by metal-organic frameworks and covalent-organic frameworks from water. *Coor*. Chem. Rev..

[41] Zhang JC (2019). Diaminomaleonitrile functionalized double-shelled hollow MIL-101 (Cr) for selective removal of uranium from simulated seawater. Chem. Eng. J..

[42] Liu R, Zhang W, Chen YT, Wang YS (2020). Uranium (VI) adsorption by copper and copper/iron bimetallic central MOFs. Colloid Surf. A..

[43] Niu CP (2022). A conveniently synthesized redox-active fluorescent covalent organic framework for selective detection and adsorption of uranium. J. Hazard. Mater..

[44] Xiong XH (2019). Ammoniating covalent organic framework (COF) for high-performance and selective extraction of toxic and radioactive uranium ions. Adv. Sci..

[45] Xu Y, Yu ZW, Zhang QY, Luo F (2023). Sulfonic-pendent vinylene-linked covalent organic frameworks enabling benchmark potential in advanced energy. Adv. Sci..

[46] Yuan YH (2021). Selective extraction of uranium from seawater with biofouling-resistant polymeric peptide. Nat. Sustain..

[47] Yang LS (2022). Bioinspired hierarchical porous membrane for efficient uranium extraction from seawater. Nat. Sustain..

[48] Wang YL (2015). Umbellate distortions of the uranyl coordination environment result in a stable and porous polycatenated framework that can effectively remove cesium from aqueous solutions. J. Am. Chem. Soc..

[49] Zhang HL (2023). Ultrafiltration separation of Am(VI)-polyoxometalate from lanthanides. Nature.

[50] Wang YX (2018). Emergence of uranium as a distinct metal center for building intrinsic X-ray scintillators. Angew. Chem. Int. Ed..

