# Supplementary material for: Ultra-selective uranium separation by in-situ formation of π-f conjugated 2D uranium-organic framework
Source: Nat Commun. 2024 Jan 11;15:453. doi: 10.1038/s41467-023-44663-4 (PMC10784586; doi:10.1038/s41467-023-44663-4)
Supplement: Supplementary file 1 — Supplementary Information [file 41467_2023_44663_MOESM1_ESM.pdf]

Supplementary Information

Ultra-selective uranium separation by *in-situ* formation of *π-f* conjugated 2D uranium-organic framework

Qing Yun Zhang,[1] Lin Juan Zhang,[2] Jian Qiu Zhu,[2] Le Le Gong,[3] Zhe Cheng Huang,[1] Feng Gao,[1] Jian Qiang Wang,[2] Xian Qing Xie,[4] and Feng Luo[1]*

[1]School of Chemistry and Materials Science, East China University of Technology, Nanchang 330013, China

[2]Key Laboratory of Interfacial Physics and Technology, Shanghai Institute of Applied Physics, Chinese Academy of Sciences, Shanghai 201800, China

[3]State Key Laboratory of NBC Protection for Civilian, Beijing 100191, China

[4]National Engineering Research Center for Carbonhydrate Synthesis, Jiangxi Normal University, Nanchang, 330027, China

E-mail: ecitluofeng@163.com

Table of contents

Uranium adsorption studies……………………………………………………………………3

Theoretical calculation…………………………………………………………………………4

X-ray absorption near-edge structure (XANES) ……………………………………………4

Supplementary Fig. 1 ……………………………………………………………………………5

Supplementary Fig. 2 ……………………………………………………………………………6

Supplementary Fig. 3 ……………………………………………………………………………7

Supplementary Fig. 4 ……………………………………………………………………………8

Supplementary Fig. 5 ……………………………………………………………………………9

Supplementary Fig. 6 ……………………………………………………………………………10

Supplementary Fig. 7 ……………………………………………………………………………11

Supplementary Fig. 8 ……………………………………………………………………………12

Supplementary Fig. 9 ……………………………………………………………………………13

Supplementary Fig. 10 …………………………………………………………………………14

Supplementary Fig. 11 …………………………………………………………………………15

Supplementary Fig. 12 …………………………………………………………………………16

Supplementary Fig. 13 …………………………………………………………………………17

Supplementary Fig. 14 …………………………………………………………………………18

Supplementary Table 1 …………………………………………………………………………19

Supplementary Table 2 …………………………………………………………………………20

Supplementary Table 3 …………………………………………………………………………21

Supplementary Table 4 …………………………………………………………………………22

Supplementary References ……………………………………………………………………23

1. Uranium adsorption studies

Uranium mother solution (1000 ppm) was prepared by dissolving $UO_2(NO_3)_2$ in a deionized water. Lower concentration solutions were prepared by diluting this solution. Adsorption temperature was 298 K. *Uranium has low radioactivity, thus we must be careful for such experiments under special protection.*

Uranium adsorption kinetics

In kinetics experiments, the Pd solution with initial concentration of 50 ppm or 1 ppm was used. The dose of adsorbent is 10 mg, while uranium solution is 10 mL. 1 M HNO_3 was used to adjust pH value of 3.

Uranium adsorption capacity

Uranium solution with initial concentration of 10-1000 ppm was used. The dose of adsorbent is 10 mg, while uranium solution is 10 mL and the contact time is 5 min. 1 M HNO_3 was used to adjust pH value of 3.

The adsorption amount, Q_e (mg/g), was calculated by the difference of uranium equilibrium concentration before and after adsorption (see equation 1):

$$Q_e = \frac{(C_0 - C_e) \times V}{m} \qquad (1)$$

where c_0 (mg/L) and c_e (mg/L) are the initial concentration and equilibrium concentration of uranium in the solutions, respectively; V (mL) is the volume of testing solution and m (mg) is the amount of sorbent.

Uranium adsorption at different acid

Uranium solution with initial concentration of 50 ppm was used. The dose of adsorbent is 10 mg, while uranium solution is 10 mL and the contact time is 5 min. 1 M HNO_3 and 1 M Na_2CO_3 was used to adjust pH value.

Ion interference experiment

In the binary ion system, the initial concentration of 10 ppm for uranium ion and 100 ppm or 1000 ppm for other ions was used. The dose of adsorbent is 10 mg, while solution is 10 mL. 1 M HNO_3 was used to adjust pH value of 3.

In the 21-ions system, the initial concentration of 10 ppm for uranium ion and other ions was used. The dose of adsorbent is 10 mg, while solution is 10 mL. 1 M HNO_3 was used to adjust pH value of 3.

Extraction of uranium from natural seawater

Natural seawater from Zhuhai, China, was used. The uranium concentration is 3.3 ppb. The dose of adsorbent is 10 mg, while the volume of natural seawater is 10 L. 1 M HNO_3 was used to adjust pH value of 3. Contacting time is 5 days.

Liquid-extraction route

The test conditions were 10 mg adsorbent dissolved in 3 mL organic solvents, 3 mL 50 ppm U solution.

2. Theoretical calculation

All first-principle calculations were performed within the framework of density functional theory (DFT) as implemented in the plane wave set Vienna *Ab-initio* Simulation Package (VASP) code[1]. The exchange-correlation terms of the correlation functions were exchanged based on the generalized gradient approximation of Perdew-Burke-Ernzerhof (GGA-PBE)[2]. A periodically projected plane wave base set of the projector enhanced wave (PAW) was employed to calculate the electron-ion interaction[3]. Spin polarization were considered in all simulations[4]. Wave functions were expanded using a plane-wave basis set with kinetic energy cutoff of 500 eV. The convergence criterion of self-consistent iteration and the ion relaxation were set at 1×10^{-4} eV and 0.02 eV $Å^{-1}$ to ensure the geometric configuration was sufficiently relaxed[5]. A gamma k-point mesh of 1×1×1 for the Brillioun zone sampling for structural optimization. The above parameters have been optimized until the energy change is negligible.

The formula of binding energy (ΔG) was defined:

$$\Delta G_b = G_{\mathrm{Ligand+UO_2}} - 3 \times G_{\mathrm{Ligand}} - G_{\mathrm{UO_2}} + n \times G_{\mathrm{H^+}} \qquad \text{equation (2)}$$

Where the G_{Ligand} and $G_{\mathrm{Ligand+UO2}}$ were the calculation total energy of pristine ligand or loading with UO_2. The G_{UO2} and $G_{\mathrm{H+}}$ were defaulted as the energy of uranyl ion and hydrogen ion. The n parameter represents the number of hydrion replaced by uranyl ion upon loading.

3. X-ray absorption fine structure (EXAFS)

The U L_3-edge X-ray absorption spectroscopy was collected in the transmission mode using inhouse laboratory-based X-ray absorption spectrometer. All XAFS data were analyzed using the program Demeter[6]. For all samples, the EXAFS oscillations were extracted from the normalized XAS spectra by subtracting the atomic background using a quadratic spline fit to k^2-weighted data, where k is the photoelectron wave number. The $\chi(k)$ functions were then Fourier transformed into R-space. The Fourier-transform window was in the k range 2-10 $Å^{-1}$. $UO_2(NO_3)_2{\cdot}6H_2O$ was used as reference sample.

4. Supplementary Figs. and Tables

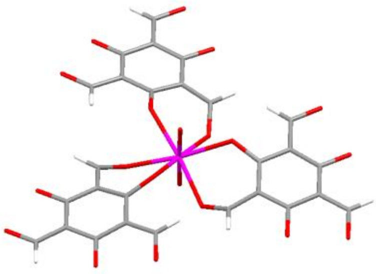

H_3TFP

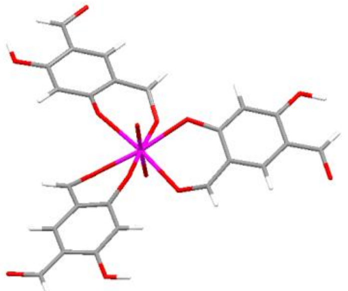

H_2HPD

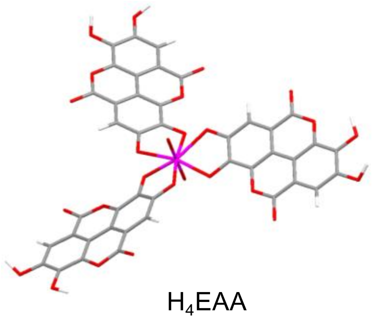

H_4EAA

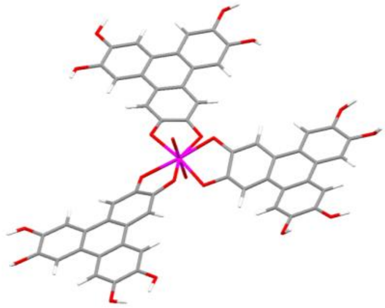

H_6HTP

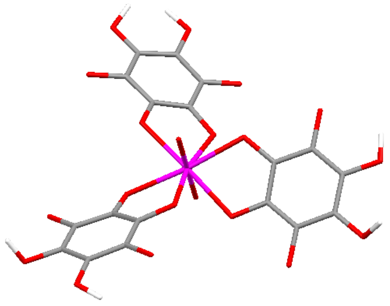

H_4THQ

Supplementary Fig. 1| View of the coordination structures between UO_2^{2+} ions and these organic ligands.

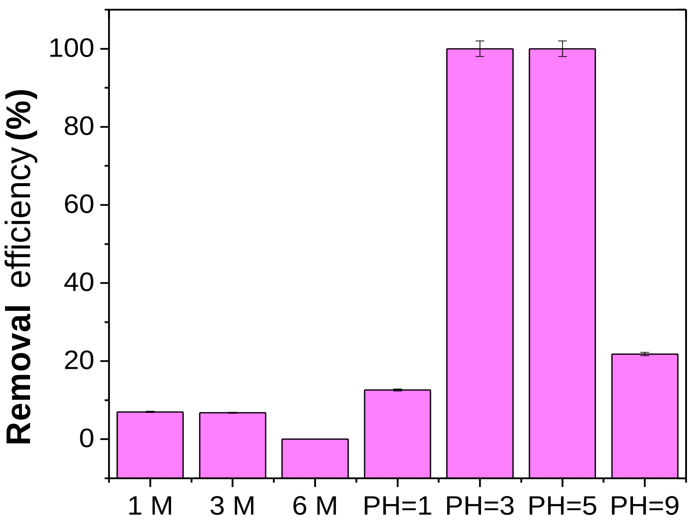

Supplementary Fig. 2| pH effect on the U adsorbent. The error bars indicate the standard deviation (n=3).

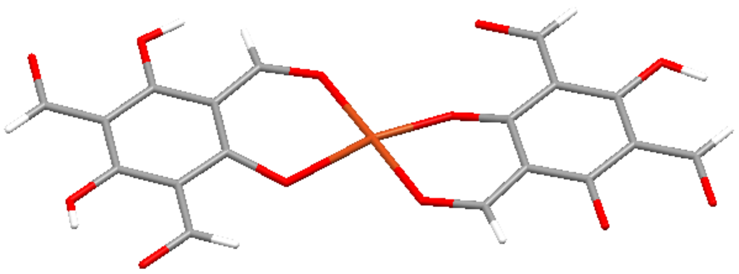

Supplementary Fig. 3| View of the optimal structure from DFT calculation for the coordination structure of Cu(II) ion with H_3TFP ligands.

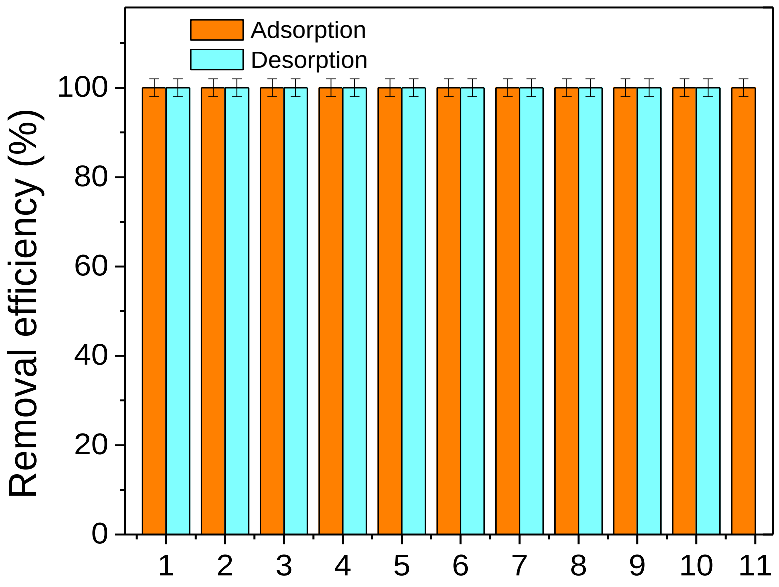

Supplementary Fig. 4| Recycle use of TFP adsorbent for U uptake. The error bars indicate the standard deviation (n=3).

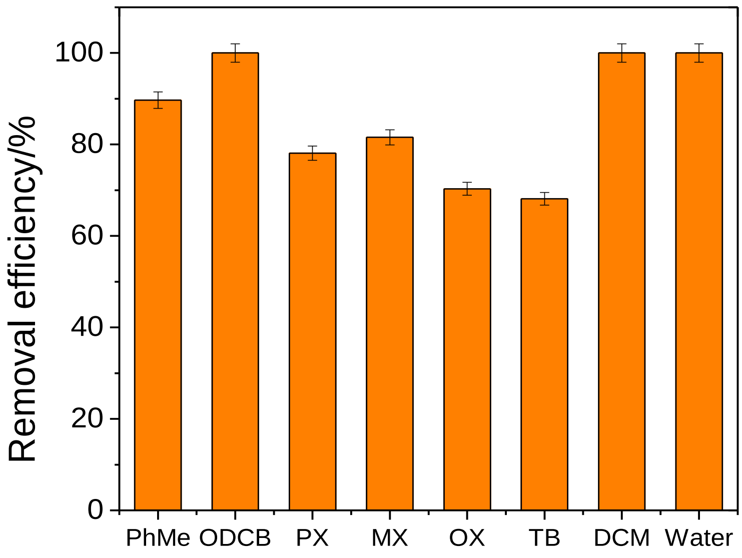

Supplementary Fig. 5| A comparison of U removal among liquid-extraction route under various organic solvents and solid-extraction route. The error bars indicate the standard deviation (n=3).

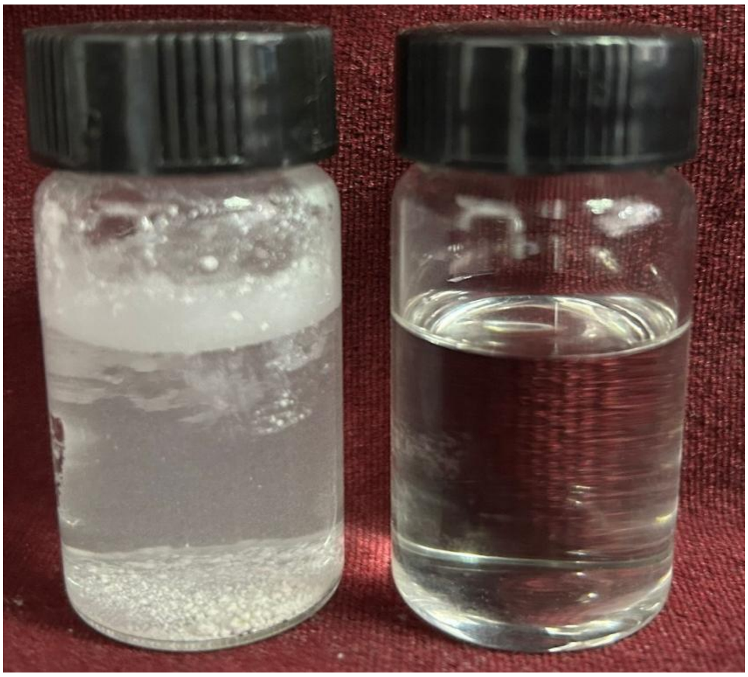

Supplementary Fig. 6| A comparison of the solution of H_3TFP in water and ODCM. Clearly, H_3TFP is insoluble in water, but completely soluble in ODCM.

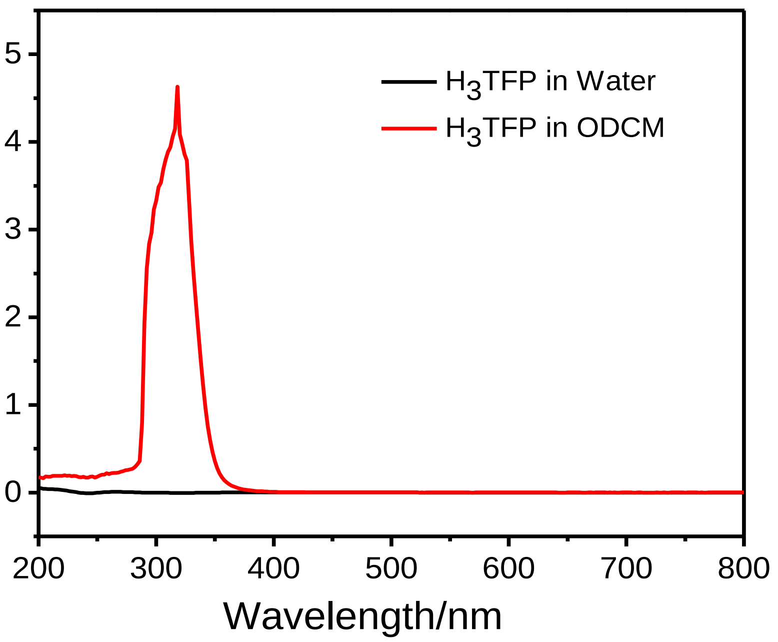

Supplementary Fig. 7| A comparison of UV-visible spectral for H_3TFP in water and ODCM. This also clearly suggests that H_3TFP is insoluble in water, but soluble in ODCM.

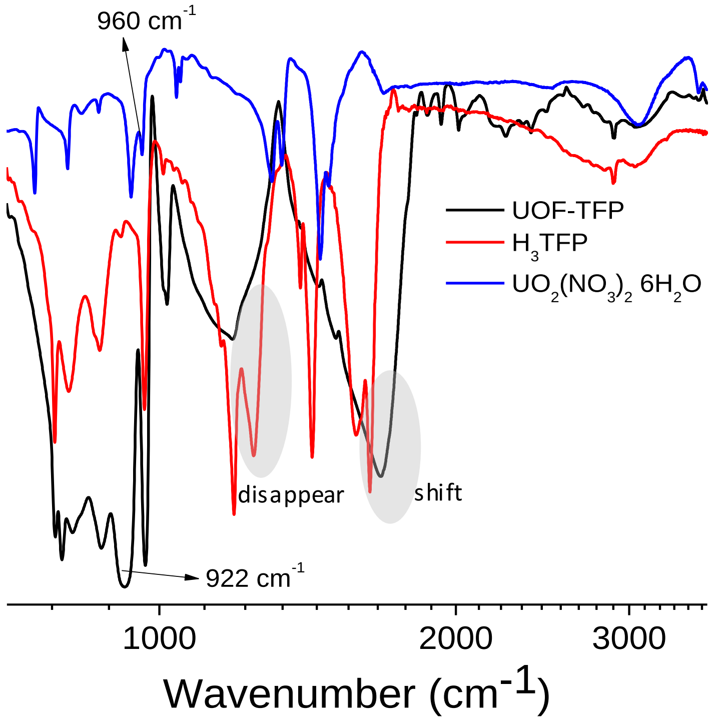

Supplementary Fig. 8| IR spectra of UOF-TFP, H_3TFP ligand, and $UO_2(NO_3)_2 \cdot 6H_2O$.

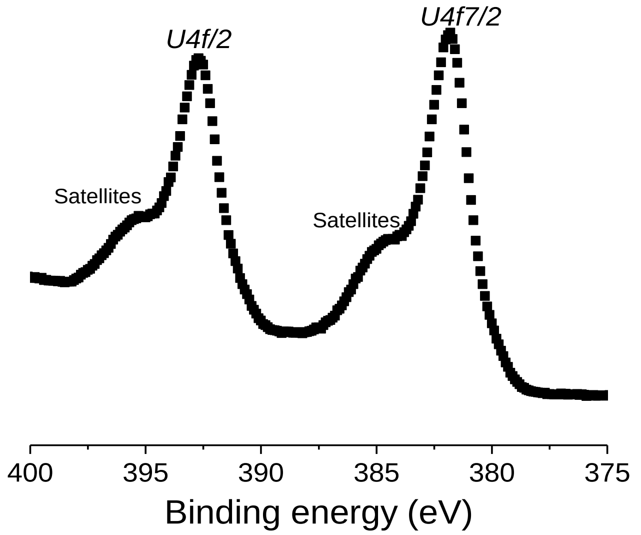

Supplementary Fig. 9| XPS spectrum of U element of UOF-TFP.

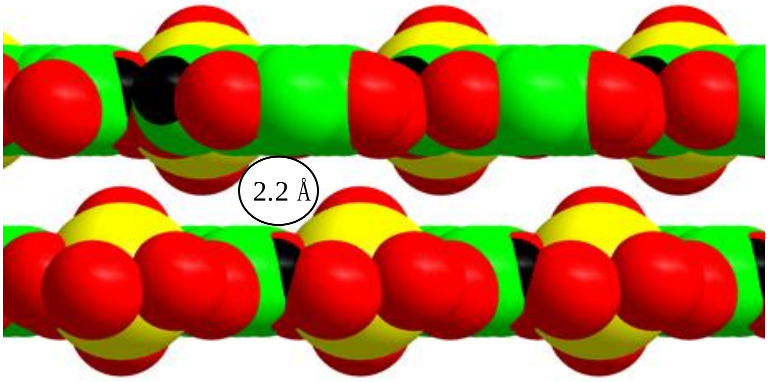

Supplementary Fig. 10| View of the small void in UOF-TFP.

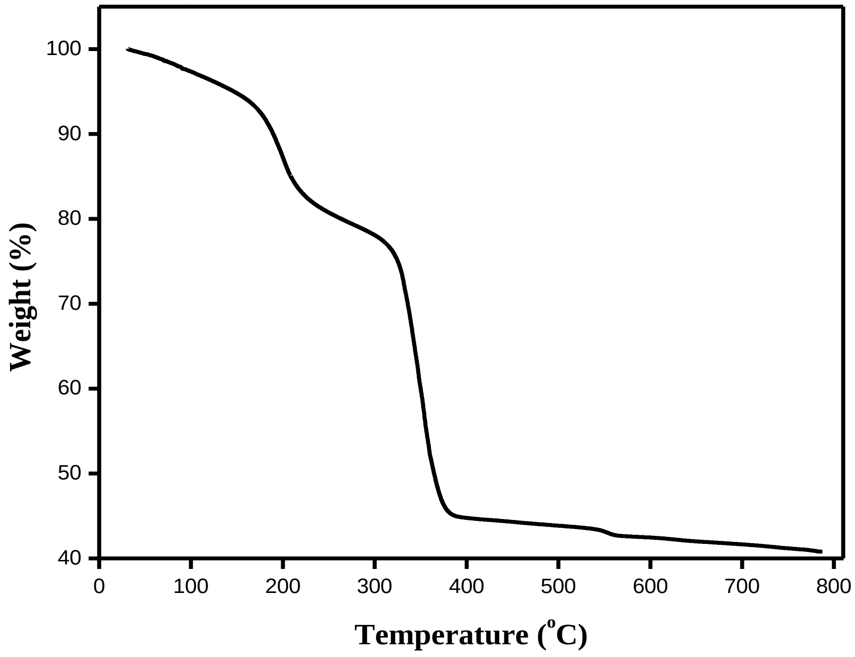

Supplementary Fig. 11| The TG plot of UOF-TFP.

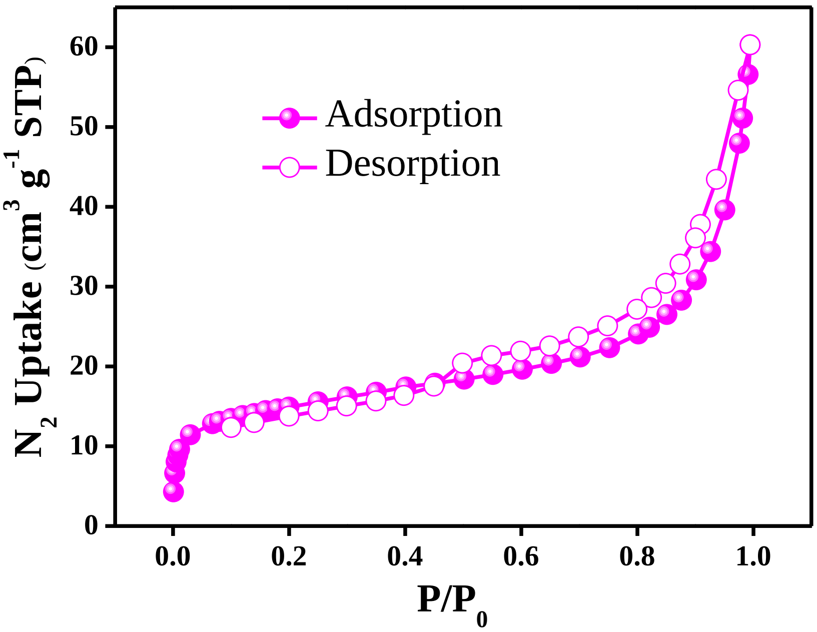

Supplementary Fig. 12| N_2 adsorption at 77 K of UOF-TFP.

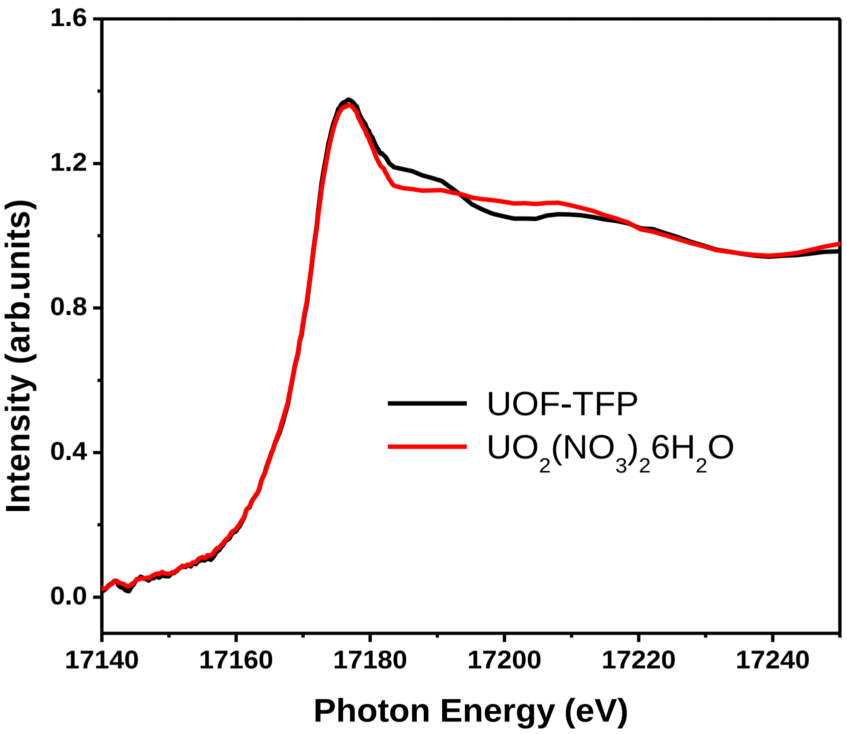

Supplementary Fig. 13| XANES of UOF-TFP and $UO_2(NO_3)_2 \cdot 6H_2O$.

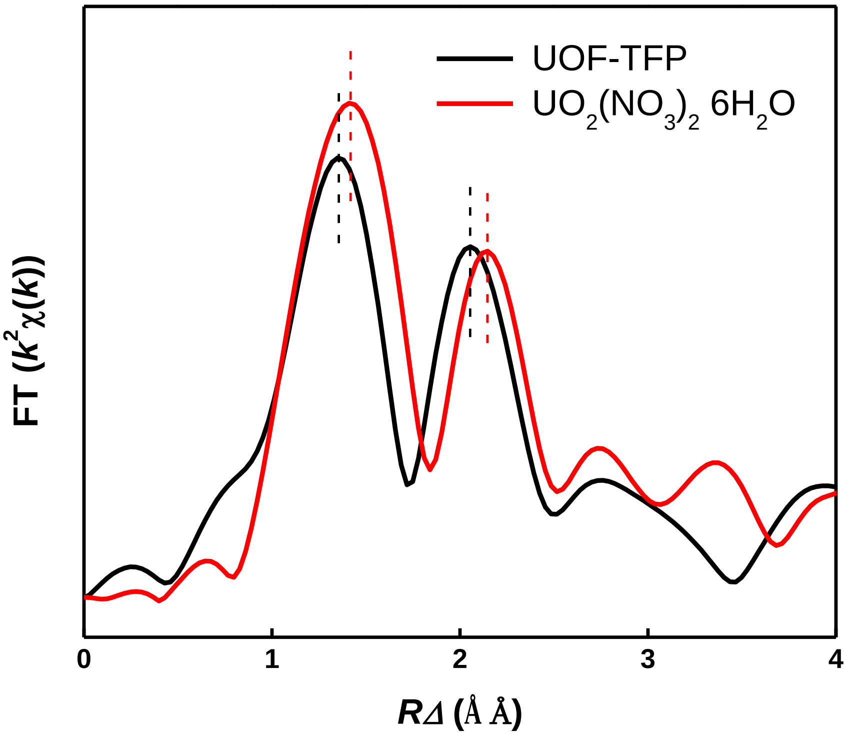

Supplementary Fig. 14| EXAFS of UOF-TFP and $UO_2(NO_3)_2 \cdot 6H_2O$.

Supplementary Table 1. A comparison in UO_2^{2+} uptake capacity among established adsorbents and our case of UOF-TFP.

Adsorbents	Adsorption capacity (mg/g)	References
TFP	1600	This work
POP_3-AO	1070	7
1-L	567	8
SCU-19	557	9
MOF-76	298	10
MOF-5	237	11
UiO-66-NH_2	114	12
Phosphorylurea-derived UiO-68	217	13
Fe_3O_4@ZIF-8	523	14
MIL-101-DETA	350	15
Cu-BTC	617	16
PCN-222-PA	401	17
ZIF-90-OM	482	18
Er-MOF	515	19
SZ-2/SZ-3	58	20
MISS-PAF-1	80	21
PIDO NF	860	22
COF-TpAb-AO	127	23
POP-oNH_2-AO	530	24
MIPAF-11c	15	25
PAF-1-NH$(CH_2)_2$AO	385	26
TFPPy-BDOH	980	27
COF-DBS	622	28
$[NH_4]^+[COF\text{-}SO_3^-]$	851	29

Supplementary Table 2. A comparison in U uptake capacity *per* day from natural seawater among established top adsorbents and our case.

Adsorbents	Time (days)	Adsorption capacity ($mg\cdot g^{-1}$)	Average adsorption capacity ($mg\cdot g^{-1}\cdot d^{-1}$)	References
TFP	5	3.2	0.64	This work
PPH-OP	21	7.63	0.36	30
AO-PIM-1	28	9.03	0.32	31
MIGPAF-13	56	16	0.28	32
POP1-AO	56	8.4	0.15	7
TFCH	30	17.9	0.59	33

Supplementary Table 3. A comparison in U uptake capacity under long time from natural seawater among established top adsorbents and our case.

Materials	Adsorbent dosage	Time (d)	Capacity for U (mg/g)	Ref.
H_3TFP	5 mg sample	10	6.4	This work
COF-HHTF-AO	5 mg sample	25	5.25	34
NDA-TN-AO	5 mg sample	27	5.94	35
Ui0-66-3C4N	20 mg sample	28	6.72	36
Anti-UiO-66	5 mg sample	30	4.5	37
PPA@MISS-PAF-1	-		0.38	38
AO-PIM-1	10 mg sample	28	8.96	31
POP1-AO	2.5 mg sample	56	8.4	7
PAF-CS	5 mg sample	21	5.88	39
MISS-PAF-1	5 mg sample	56	5.6	21
POP-oNH_2- AO	5 mg sample	56	3.92	24
P(2DVB-VBC)-2PAN	10 mg sample	27	1.89	40
AO-HNTs	10 mg sample	30	9	41
MIGPAF-13	4 mg sample	28	7.84	32
Fe-Nx-C-R	6 mg sample	1	1.20	42
Cp-1:12	10 mg sample	28	5.6	43
SMON-PAO	10 mg sample	56	9.52	44
Zn^{2+}-PAO	36 mg sample	28	9.24	45
PPH-OP	10 mg sample	21	7.12	30
DNA-UEH	10 mg sample	6	6.0	46
MS@PIDO/Alg sponge	-	56	5.6	47

'-' represents the data that can not be obtained from reports.

Supplementary Table 4. A comparison in U uptake capacity under long time from natural seawater among established top adsorbents and our case.

Pair	CN	R(Å)	$\sigma^2 \times 10^{-3}$ (Å^{-3})
U-O_{ax}	2.6±0.3	1.85±0.01	1.0
U-O_{eq1}	2.8±0.6	2.42±0.02	3.5
U-O_{eq2}	3.0±1.3	2.51±0.01	1.0

R is the coodination bond length, CN is the coordination number, and σ^2 is the Debye-Waller (disorder factor). U-O_{ax} represents the U=O coordination bonds, U-O_{eq1} represents the U-$O_{hydroxyl}$ bond and U-O_{eq2} represents U-$O_{aldehyde}$ bond.

Supplementary Reference

1. Kresse, G. & Furthmüller, J. Efficient iterative schemes for ab initio total-energy calculations using a plane-wave basis set. *Phys. Rev. B* **54**, 11169-11186 (1996).
2. Perdew, J. P., Burke, K. & Ernzerhof, M. Generalized Gradient Approximation Made Simple. *Phys. Rev. Lett.* **77**, 3865-3868 (1996).
3. Solovyev, I. V., Dederichs, P. H. & Anisimov, V. I. Corrected atomic limit in the local-density approximation and the electronic structure of d impurities in Rb. *Phys. Rev. B* **50**, 16861-16871 (1994).
4. Gong, L. et al. Catalytic mechanism and design principle of coordinately unsaturated single metal atom-doped covalent triazine frameworks with high activity and selectivity for CO_2 electroreduction. *J. Mater. Chem. A.* **9**, 3555-3566 (2021).
5. Gong, L. et al. Catalytic mechanisms and design principles for single-atom catalysts in highly efficient CO_2 conversion. *Adv. Energy Mater.* **9**, 1902625 (2019).
6. Ravel, B. & Newville, M. ATHENA, ARTEMIS, HEPHAESTUS: data analysis for X-ray absorption spectroscopy using IFEFFIT. *J. Synchrotron Rad.* **12**, 537-541 (2005).
7. Song, Y. et al. Nanospace decoration with uranyl-specific "Hooks" for selective uranium extraction from seawater with ultrahigh enrichment index. *ACS Cent. Sci.* **7**, 1650-1656 (2021).
8. Wang, X.-F. et al. Cooperative capture of uranyl ions by a carbonyl-bearing hierarchical-porous Cu-organic framework. *Angew. Chem. Int. Ed.* **58**, 18808-18812 (2019)
9. Zhang, H. et al. Three mechanisms in one material: uranium capture by a polyoxometalate-organic framework through combined complexation, chemical reduction, and photocatalytic reduction. *Angew. Chem. Int. Ed.* **58**, 16110-16114 (2019)
10. Yang, W. et al. MOF-76: from a luminescent probe to highly efficient U(VI) sorption material. *Chem. Commun.* **49**, 10415-10417 (2013).
11. Wu, Y. et al. Synthesis of rod-like metal-organic framework (MOF-5) nanomaterial for efficient removal of U(VI): batch experiments and spectroscopy study. *Sci. Bull.* **63**, 831-839 (2018).
12. Luo, B.-C., Yuan, L.-Y., Chai, Z.-F., Shi, W.-Q. & Tang, Q. U(VI) capture from aqueous solution by highly porous and stable MOFs: UiO-66 and its amine derivative. *J. Radioanal. Nucl. Chem.* **307**, 269-276 (2016).
13. Carboni, M., Abney, C. W., Liu, S. & Lin, W. Highly porous and stable metal-organic frameworks for uranium extraction. *Chem. Sci.* **4**, 2396-2402 (2013).
14. Min, X. et al. Fe_3O_4@ZIF-8: a magnetic nanocomposite for highly efficient UO_2^{2+} adsorption and selective UO_2^{2+}/Ln^{3+} separation. *Chem. Commun.* **53**, 4199-4202 (2017).
15. Bai, Z.-Q. et al. Introduction of amino groups into acid-resistant MOFs for enhanced U(VI) sorption. *J. Mater. Chem. A.* **3**, 525-534 (2015).
16. Liu, R., Zhang, W., Chen, Y. & Wang, Y. Uranium (VI) adsorption by copper and copper/iron bimetallic central MOFs. *Colloid. Surface. A* **587**, 124334 (2020).
17. Peng, Y., Zhang, Y., Tan, Q. & Huang, H. Bioinspired construction of uranium ion trap with abundant phosphate functional groups. *ACS Appl. Mater. Inter.* **13**, 27049-27056 (2021).
18. Mei, D. et al. Efficient uranium adsorbent with antimicrobial function: oxime functionalized ZIF-90. *Chem. Eng. J.* **425**, 130468 (2021).
19. Zhang, Z.-H. et al. Rational construction of porous metal-organic frameworks for uranium(VI) extraction: the strong periodic tendency with a metal node. *ACS Appl. Mater. Inter.* **12**,

14087-14094 (2020).
20. Zheng, T. et al. Overcoming the crystallization and designability issues in the ultrastable zirconium phosphonate framework system. *Nat. Commun.* **8**, 15369 (2017).
21. Yuan, Y. et al. A molecular coordination template strategy for designing selective porous aromatic framework materials for uranyl capture. *ACS Central Science* **5**, 1432-1439 (2019).
22. Wang, D. et al. Significantly enhanced uranium extraction from seawater with mass produced fully amidoximated nanofiber adsorbent. *Adv. Energy Mater.* **8**, 1802607 (2018).
23. Sun, Q. et al. Covalent organic frameworks as a decorating platform for utilization and affinity enhancement of chelating sites for radionuclide sequestration. *Adv. Mater.* **30**, 1705479 (2018).
24. Sun, Q. et al. Bio-inspired nano-traps for uranium extraction from seawater and recovery from nuclear waste. *Nat. Commun.* **9**, 1644 (2018).
25. Yuan, Y. et al. Molecularly imprinted porous aromatic frameworks and their composite components for selective extraction of uranium ions. *Adv. Mater.* **30**, 1706507 (2018).
26. Aguila, B. et al. Design strategies to enhance amidoxime chelators for uranium recovery. *ACS Appl. Mater. Interf.* **11**, 30919-30926 (2019).
27. Li, Z. et al. Functionalized polyarylether-based COFs for rapid and selective extraction of uranium from aqueous solution. *Chem. Eng. J.* **434**, 134623 (2022).
28. Xu, Y., Yu, Z., Zhang, Q. & Luo, F. Sulfonic-pendent vinylene-linked covalent organic frameworks enabling benchmark potential in advanced energy. *Adv. Sci.* **10**, 2300408 (2023).
29. Xiong, X. H. et al. Ammoniating covalent organic framework (COF) for high-performance and selective extraction of toxic and radioactive uranium Ions. *Adv. Sci.* **6**, 1900547 (2019).
30. Yuan, Y. et al. Selective extraction of uranium from seawater with biofouling-resistant polymeric peptide. *Nat. Sustain.* **4**, 708-714 (2021).
31. Yang, L. et al. Bioinspired hierarchical porous membrane for efficient uranium extraction from seawater. *Nat. Sustain.* **5**, 71-80 (2022).
32. Wang, Z. et al. Constructing uranyl-specific nanofluidic channels for unipolar ionic transport to realize ultrafast uranium extraction. *J. Am. Chem. Soc.* **143**, 14523-14529 (2021).
33. Kaushik, A. et al. Large-area self-standing thin film of porous hydrogen-bonded organic framework for efficient uranium extraction from seawater. *Chem* **8**, 2749-2765 (2022).
34. Cheng, G. et al. Extremely stable amidoxime functionalized covalent organic frameworks for uranium extraction from seawater with high efficiency and selectivity. *Sci. Bull.* **66**, 1994-2001 (2021).
35. Cui, W.-R. et al. Regenerable covalent organic frameworks for photo-enhanced uranium adsorption from seawater. *Angew. Chem. Int. Ed.* **59**, 17684-17690 (2020).
36. Yuan, Y. et al. A bio-inspired nano-pocket spatial structure for targeting uranyl capture. *Angew. Chem. Int. Ed.* **59**, 4262-4268 (2020).
37. Yu, Q. et al. A universally applicable strategy for construction of anti-biofouling adsorbents for enhanced uranium recovery from seawater. *Adv. Sci.* **6**, 1900002 (2019).
38. Wang, Z. et al. Constructing an ion pathway for uranium extraction from seawater. *Chem* **6**, 1683-1691 (2020).
39. Li, Z. et al. Constructing amidoxime-modified porous adsorbents with open architecture for cost-effective and efficient uranium extraction. *Chem. Sci.* **11**, 4747-4752 (2020).
40. Yue, Y. et al. Seawater uranium sorbents: preparation from a mesoporous copolymer initiator by atom-transfer radical polymerization. *Angew. Chem. Int. Ed.* **52**, 13458-13462 (2013).

41. Zhao, S. et al. A dual-surface amidoximated halloysite nanotube for high-efficiency economical uranium extraction from seawater. *Angew. Chem. Int. Ed.* **58**, 14979-14985 (2019).
42. Yang, H. et al. Functionalized iron-nitrogen-carbon electrocatalyst provides a reversible electron transfer platform for efficient uranium extraction from seawater. *Adv. Mater.* **33**, 2106621 (2021).
43. Yue, Y. et al. Polymer-coated nanoporous carbons for trace seawater uranium adsorption. *Sci. China Chem.* **56**, 1510-1515 (2013).
44. Yuan, Y. et al. Rational design of porous nanofiber adsorbent by blow-spinning with ultrahigh uranium recovery capacity from seawater. *Adv. Funct. Mater.* **29**, 1805380 (2019).
45. Yan, B., Ma, C., Gao, J., Yuan, Y. & Wang, N. An ion-crosslinked supramolecular hydrogel for ultrahigh and fast uranium recovery from seawater. *Adv. Mater.* **32**, 1906615 (2020).
46. Yuan, Y. et al. DNA nano-pocket for ultra-selective uranyl extraction from seawater. *Nat. Commun.* **11**, 5708 (2020).
47. Wang, D. et al. A marine-inspired hybrid sponge for highly efficient uranium extraction from seawater. *Adv. Funct. Mater.* **29**, 1901009 (2019).
