# Supplementary material for: Ultra-selective uranium separation by in-situ formation of π-f conjugated 2D uranium-organic framework
Source: Nat Commun. 2024 Jan 11;15:453. doi: 10.1038/s41467-023-44663-4 (PMC10784586; doi:10.1038/s41467-023-44663-4)
Supplement: Supplementary file 2 — Peer review file [file 41467_2023_44663_MOESM2_ESM.pdf]

nature portfolio

Peer Review File

Ultra-selective uranium separation by in-situ formation of π-f conjugated 2D uranium-organic framework

Open Access This file is licensed under a Creative Commons Attribution 4.0 International License, which permits use, sharing, adaptation, distribution and reproduction in any medium or format, as long as you give appropriate credit to the original author(s) and the source, provide a link to the Creative Commons license, and indicate if changes were made. In the cases where the authors are anonymous, such as is the case for the reports of anonymous peer reviewers, author attribution should be to 'Anonymous Referee' followed by a clear attribution to the source work. The images or other third party material in this file are included in the article's Creative Commons license, unless indicated otherwise in a credit line to the material. If material is not included in the article's Creative Commons license and your intended use is not permitted by statutory regulation or exceeds the permitted use, you will need to obtain permission directly from the copyright holder. To view a copy of this license, visit http://creativecommons.org/licenses/by/4.0/.

REVIEWER COMMENTS

Reviewer #1 (Remarks to the Author):

For achieving uranium separation, different from the established methods relying on MOFs, COF, or POPs, the authors demonstrated in this work a facile approach of UOF-based approach using just an organic ligand. The results are very appealing, such as uranium theoretical uptake up to 1.6 g/g, high uranium capture capacity of 0.64 mg·g-1·d-1 from natural seawater, and high U selectivity over other metal ions. All these merits come from the unique design for such uranium separation by means of the in-situ formation of π-f conjugated 2D uranium-organic framework. This reviewer supports its publication in Nature Comm but revisions are needed to address the comments below.
1. The authors used five comparable ligands to fix uranium ions, and difference in the uranium uptake was obtained. However, no reason was given for such difference. It is suggested DFT calculations be performed to explain the difference, which could be instructive for new design.
2. Although the structure of UOF is clear and some strong evidences have been shown; however, additional evidences such as EXAFS are needed to further verify the structure of such UOF.
3. The author emphasized the advantage of such UOF method. How’s the cost for uranium capture from seawater as compared with some established methods?
4. References are needed for some comparisons in the manuscript, e.g. page 9, line 213.
5. Units should be unified, for example in Fig. 3, ppm and mg/L are mixed.

Reviewer #2 (Remarks to the Author):

Uranium separation based on adsorption technology has always been a hot topic and challenge in current research. For example, there has developed several remarkable adsorbents, such as porous polymers (POPs), metal-organic frameworks (MOFs), and covalent organic frameworks (COFs). As stated by the author, these established adsorbents indeed need the first preparation of such materials, and next uranium separation can be executed. By contrast, the author in this work showed a distinct method with the just use of small functionalized organic molecule, 1,3,5-triformylphloroglucinol, but they can achieve a big outcome in uranium separation through an uncommon precipitation method. Whatever the adsorption capacity, adsorption rate, adsorption selectivity, as well as reuse of adsorbents and uranium extraction from seawater, all these indicate its advantage over current adsorbents. The mechanism is also clear and contributed by the in-situ formation of π-f conjugated 2D uranium-organic framework. Although I really appreciate this job, however, I think it still needs to be improved as follows.
There is no problem using 1,3,5-triformylphloroglucinol as an adsorbent. However, there lacks the data to support the solubility of 1,3,5-triformylphloroglucinol in the water. This is very important for such MOF-based route.
The U selectivity over Cu^{2+}, Fe^{3+}, Nd^{3+}, Th^{4+}, and VO_3^- ions are attractive. But there lack in-depth discussion in the reason. Especially for Cu^{2+} ions, it can also form d-f conjugated 2D Cu-organic framework.
Such precipitation method is interesting and effective. However, in light of the nature of 1,3,5-triformylphloroglucinol, can the author extend to liquid extraction by 1,3,5-triformylphloroglucinol? This will be also attractive for uranium separation, if the results are positive.
The current uranium extraction from seawater is exciting. However, I still concern about its adsorption capacity after one month, since the current adsorption capacity is till low, relative to other benchmark adsorbents.
Minor issues
The title is long. ‘Ultra-selective uranium separation by in-situ formation of π-f conjugated 2D uranium-organic framework’ is more proper.
Page 1, line 9, ‘has’ should be ‘have’. Please also check other grammar and spelling errors.
Page 6, there lacks a discussion to explain the pH effect.
Page 9, IR of $UO_2(NO_3)_2·6H_2O$ is suggested to be tested.

Reviewer #3 (Remarks to the Author):

This work reported a unique route for uranium separation by means of a precipitation method. Interestingly, such precipitation method not only afforded ultrahigh uranium selectivity over other ions, but also high adsorption capacity, fast adsorption kinetics, high affinity, and good reusability. All these features confirmed its superiority over common adsorption method. Subsequently, upon such precipitation method, the author also observed outstanding performance in extraction of uranium from seawater. Based on these characterizations including in IR, XPS, PXRD, SEM-EDS, and TEM, the author proposed a mechanism of in-situ formation of π-f conjugated 2D uranium-organic framework (UOF). These results are exciting and preparation was carried out carefully. The manuscript is clearly written and well organized. Considering its novelty and the outstanding performance for uranium extraction, I consider this manuscript is appropriate for the Nature Communications after minor revision.
For the content of the manuscript, I suggest that the author strengthen the exploration of the mechanism, e. g. (1) why is H3TFP ligand the best? what is relationship between uranium capture and organic ligand? who is the key factor? (2) why is the uranium selectivity better over other ions using H3TFP ligand? (3) Assuming such precipitation method with this mechanism of in-situ formation of π-f conjugated 2D UOF, however, we are not clear about the coordination number and bond length, which calls additional characterizations in this aspect.

Reviewers' Comments:

Reviewer #1 (Remarks to the Author):

For achieving uranium separation, different from the established methods relying on MOFs, COF, or POPs, the authors demonstrated in this work a facile approach of UOF-based approach using just an organic ligand. The results are very appealing, such as uranium theoretical uptake up to 1.6 g/g, high uranium capture capacity of 0.64 mg·g-1·d-1 from natural seawater, and high U selectivity over other metal ions. All these merits come from the unique design for such uranium separation by means of the in-situ formation of π-f conjugated 2D uranium-organic framework. This reviewer supports its publication in Nature Comm but revisions are needed to address the comments below.

1. The authors used five comparable ligands to fix uranium ions, and difference in the uranium uptake was obtained. However, no reason was given for such difference. It is suggested DFT calculations be performed to explain the difference, which could be instructive for new design.
2. Although the structure of UOF is clear and some strong evidences have been shown; however, additional evidences such as EXAFS are needed to further verify the structure of such UOF.
3. The author emphasized the advantage of such UOF method. How's the cost for uranium capture from seawater as compared with some established methods?
4. References are needed for some comparisons in the manuscript, e.g. page 9, line 213.
5. Units should be unified, for example in Fig. 3, ppm and mg/L are mixed.

Reviewer #2 (Remarks to the Author):

Uranium separation based on adsorption technology has always been a hot topic and challenge in current research. For example, there has developed several remarkable adsorbents, such as porous polymers (POPs), metal-organic frameworks (MOFs), and covalent organic frameworks (COFs). As stated by the author, these established adsorbents indeed need the first preparation of such materials, and next uranium separation can be executed. By contrast, the author in this work showed a distinct method with the just use of small functionalized organic molecule, 1,3,5-triformylphloroglucinol, but they can achieve a big outcome in uranium separation through an uncommon precipitation method. Whatever the adsorption capacity, adsorption rate, adsorption selectivity, as well as reuse of adsorbents and uranium extraction from seawater, all these indicate its advantage over current adsorbents. The mechanism is also clear and contributed by the in-situ formation of π-f conjugated 2D uranium-organic framework. Although I really appreciate this job, however, I think it still needs to be improved as follows.

There is no problem using 1,3,5-triformylphloroglucinol as an adsorbent. However, there lacks

the data to support the solubility of 1,3,5-triformylphloroglucinol in the water. This is very important for such MOF-based route.

The U selectivity over Cu2+, Fe3+, Nd3+, Th4+, and VO3- ions are attractive. But there lack in-depth discussion in the reason. Especially for Cu2+ ions, it can also form d-f conjugated 2D Cu-organic framework.

Such precipitation method is interesting and effective. However, in light of the nature of 1,3,5-triformylphloroglucinol, can the author extend to liquid extraction by 1,3,5-triformylphloroglucinol? This will be also attractive for uranium separation, if the results are positive.

The current uranium extraction from seawater is exciting. However, I still concern about its adsorption capacity after one month, since the current adsorption capacity is till low, relative to other benchmark adsorbents.

Minor issues

The title is long. ‘Ultra-selective uranium separation by in-situ formation of π-f conjugated 2D uranium-organic framework’ is more proper.

Page 1, line 9, ‘has’ should be ‘have’. Please also check other grammar and spelling errors.

Page 6, there lacks a discussion to explain the pH effect.

Page 9, IR of UO2(NO3)2·6H2O is suggested to be tested.

Reviewer #3 (Remarks to the Author):

This work reported a unique route for uranium separation by means of a precipitation method. Interestingly, such precipitation method not only afforded ultrahigh uranium selectivity over other ions, but also high adsorption capacity, fast adsorption kinetics, high affinity, and good reusability. All these features confirmed its superiority over common adsorption method. Subsequently, upon such precipitation method, the author also observed outstanding performance in extraction of uranium from seawater. Based on these characterizations including in IR, XPS, PXRD, SEM-EDS, and TEM, the author proposed a mechanism of in-situ formation of π-f conjugated 2D uranium-organic framework (UOF). These results are exciting and preparation was carried out carefully. The manuscript is clearly written and well organized. Considering its novelty and the outstanding performance for uranium extraction, I consider this manuscript is appropriate for the Nature Communications after minor revision.

For the content of the manuscript, I suggest that the author strengthen the exploration of the mechanism, e. g. (1) why is H3TFP ligand the best? what is relationship between uranium capture and organic ligand? who is the key factor? (2) why is the uranium selectivity better over other ions

using H3TFP ligand? (3) Assuming such precipitation method with this mechanism of in-situ formation of π-f conjugated 2D UOF, however, we are not clear about the coordination number and bond length, which calls additional characterizations in this aspect.

Response to reviewer #1:

Thank for your useful comments on improving the quality of our manuscript. According to your comments, we have carefully made a revised manuscript and supplementary, which are highlighted in red in the revision.

The point-to-point response is listed as follows.

Comment 1: The authors used five comparable ligands to fix uranium ions, and difference in the uranium uptake was obtained. However, no reason was given for such difference. It is suggested DFT calculations be performed to explain the difference, which could be instructive for new design.

Response: Thank you this comment. In the revision, DFT calculation was added, and the difference in uranium capture for these organic ligands was clearly explained by the binding energy, the coordination structure and coplanarity. And a discussion was added in the revision (see page 3-4, highlight in red, Supplementary Fig. 1).

Correction:

To clarify the difference in UO_2^{2+} capture for these organic ligands, we then carried out the calculation on the binding energy (ΔG) of these organic ligands with UO_2^{2+} ion by density functional theory (DFT) method. A planar six-coordination model of one UO_2^{2+} ion coordinated by three these organic ligands was used to carried out DFT calculation. The optimized coordination structures of them were shown in Supplementary Fig. 1. The binding energy (ΔG) gives a hierarchy of H_4THQ (-0.29 eV)>H_6HTP (-0.69 eV)>H_4EAA (-1.02 eV)>H_2HPD (-2.99 eV)>H_3TFP (-3.91 eV). Generally, negative binding energy (ΔG) suggests the reaction thermodynamically spontaneous, while this also obeys a rule, *viz.* the more negative, and the stronger the binding. Thus, the negative ΔG values mean that all these organic ligands can capture UO_2^{2+} ion through coordination, which is consistent with the experimental results, while the smallest and biggest ΔG value in respectively H_3TFP and H_4THQ means the strongest and weakest binding and consequently the biggest and smallest UO_2^{2+} uptake, which is also in good agreement with the experimental results. Moreover, the hierarchy in binding energy (ΔG) is also in accord with the hierarchy in the UO_2^{2+} uptake, confirming the adsorption of UO_2^{2+} ions by these organic ligand obeying the defined planar coordination principle. In addition, seen from the optimized coordination structures of them, it is found that the chelate coordination in the combination of one aldehyde oxygen and one hydroxyl oxygen is more beneficial for U-O coordination and planar coordination configuration such as H_4TFP and H_2HPD over the chelate coordination from two hydroxyl oxygens such as H_4EAA, H_6HTP, H_4THQ, due to smaller steric hindrance in the combination of one aldehyde oxygen and one hydroxyl oxygen. And this could be the key to determine the UO_2^{2+} uptake performance. Furthermore, big conjugated structure is also more beneficial for planar coordination configuration and consequently UO_2^{2+} uptake, e.g. H_6HTP and H_4EAA vs. H_4THQ.

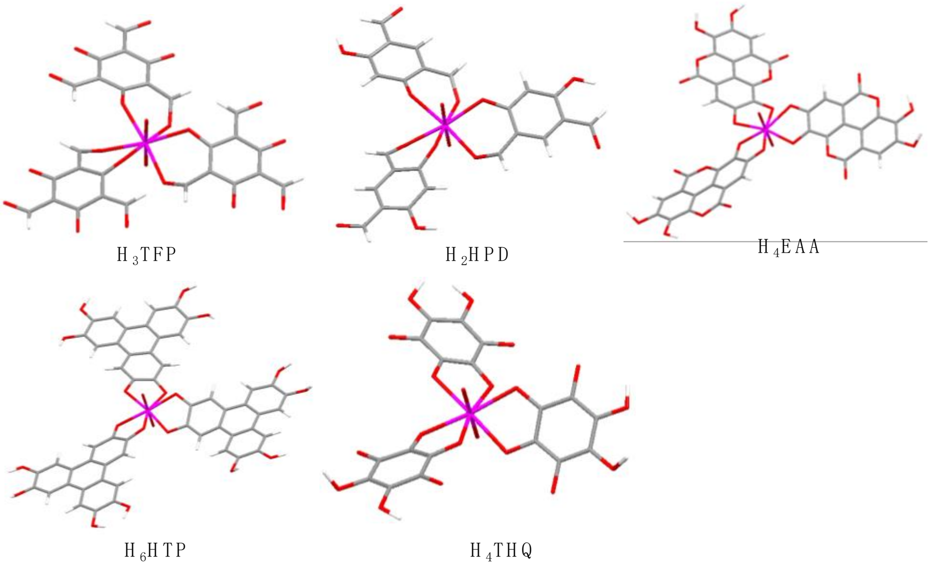

Supplementary Fig. 1| View of the coordination structures between UO_2^{2+} ions and these organic ligands.

Comment 2: Although the structure of UOF is clear and some strong evidences have been shown; however, additional evidences such as EXAFS are needed to further verify the structure of such UOF.

Response: Thank you this comment. In the revision, EXAFS was measured. The fitting results strongly suggests the planar six-coordination with two different types in the bond length of 2.42±0.02 Å and 2.51±0.01 Å (see Fig. 6 and Supplementary Figs. 13, 14, Table 4), which is in good agreement with the experimental results, thus, strongly supporting our previous results.

Correction: In addition, to gain deep insight into the local coordination sphere of the uranium species, U K-edge X-ray absorption near-edge structure (XANES, Supplementary Fig. 13) and extended X-ray absorption fine structure (EXAFS, Supplementary Fig. 14) spectroscopy were performed. $UO_2(NO_3)_2{\cdot}6H_2O$ was used as the reference standard. It was found that UOF-TFP rendered comparable XANES and EXAFS spectra with that observed in $UO_2(NO_3)_2{\cdot}6H_2O$, confirming their similarity in the valence state and coordination surrounding of uranium. As we know, uranium in $UO_2(NO_3)_2{\cdot}6H_2O$ is hexavalent in the form of UO_2^{2+}, and UO_2^{2+} in $UO_2(NO_3)_2{\cdot}6H_2O$ takes planar six coordination with two NO_3^- ions and two coordination water molecules, where each NO_3^- ion displays a chelate coordination mode with two NO_3^- oxygen atoms to fix one UO_2^{2+} ion. Accordingly, we can deduce also UO_2^{2+} ions with also the planar six coordination in UOF-TFP (Supplementary Table 4). High-quality fits of the EXAFS data for UOF-TFP (Figs. 6a and 6b) strongly suggest an eight coordination of uranium, where two U-O coordinations with bond length of 1.85±0.01 Å are assigned to U=O bond of UO_2^{2+} ion, other six U-O coordinations with longer bond length are assigned to the planar U-O coordination from TFP^{3-} ligand. This also reveals two types of U-O bond with equal numbers in the bond length of 2.42±0.02 Å and 2.51±0.01 Å, respectively. We then comparied the results from EXAFS data with the results from structural simulation, and found that the U=O bond of UO_2^{2+} ion form EXAFS data is well consistent with the results from structural simulation (1.84 Å), while the bond length of 2.42±0.02 Å and 2.51±0.01 Å from

EXAFS data is close to U-O$_{hydroxyl}$ bond (2.40 Å, hydroxyl oxygen of TFP^{3-} as coordination atom) and U-O$_{aldehyde}$ bond (2.70 Å, aldehyde oxygen of TFP^{3-} as coordination atom) from structural simulation. Thereby, the structure of UOF-TFP can be once again confirmed by EXAFS results.

(Page 12)

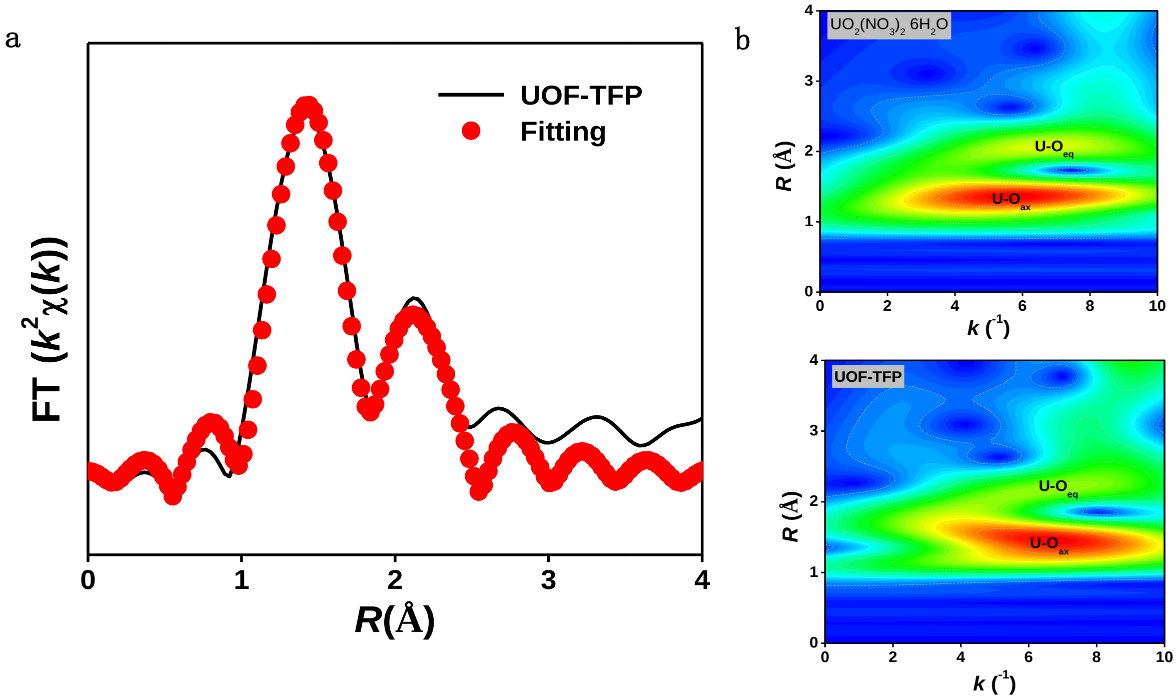

Fig. 6| EXZAF of UOF-TFP. a Experimental EXZAF data (red) and fitting results (black). **b** View of coordination surrounding of UOF-TFP and $UO_2(NO_3)_2 \cdot 6H_2O$, where U-O$_{ax}$ represents the U=O coordination bonds, U-O$_{eq}$ represents the U-O$_{nitrate}$ *plus* U-O$_{water}$ in $UO_2(NO_3)_2 \cdot 6H_2O$ and U-O$_{hydroxyl}$ *plus* U-O$_{aldehyde}$ in UOF-TFP.

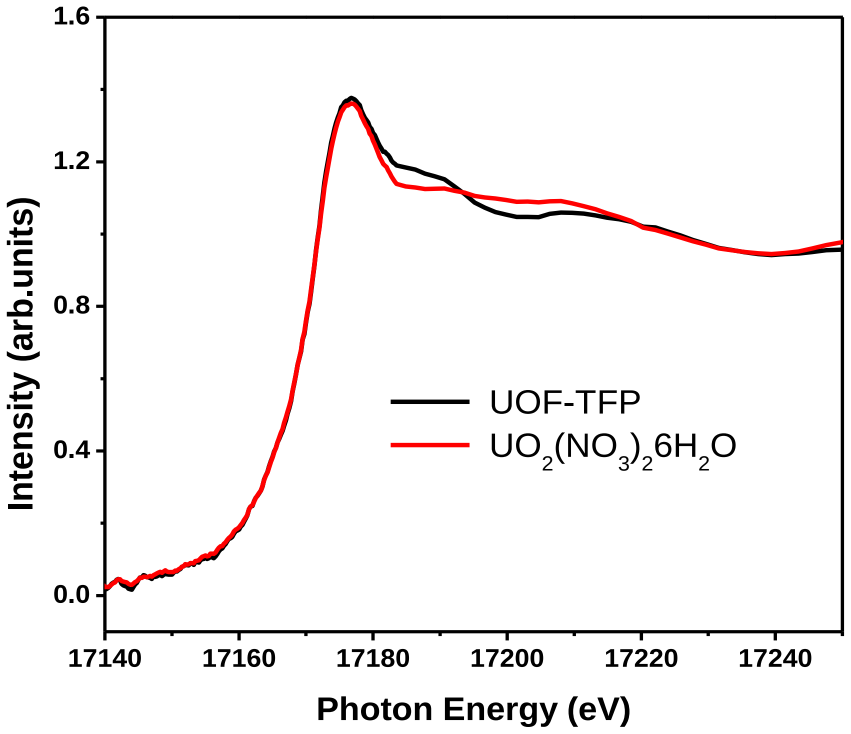

Supplementary Fig. 13| XANES of UOF-TFP and $UO_2(NO_3)_2 \cdot 6H_2O$.

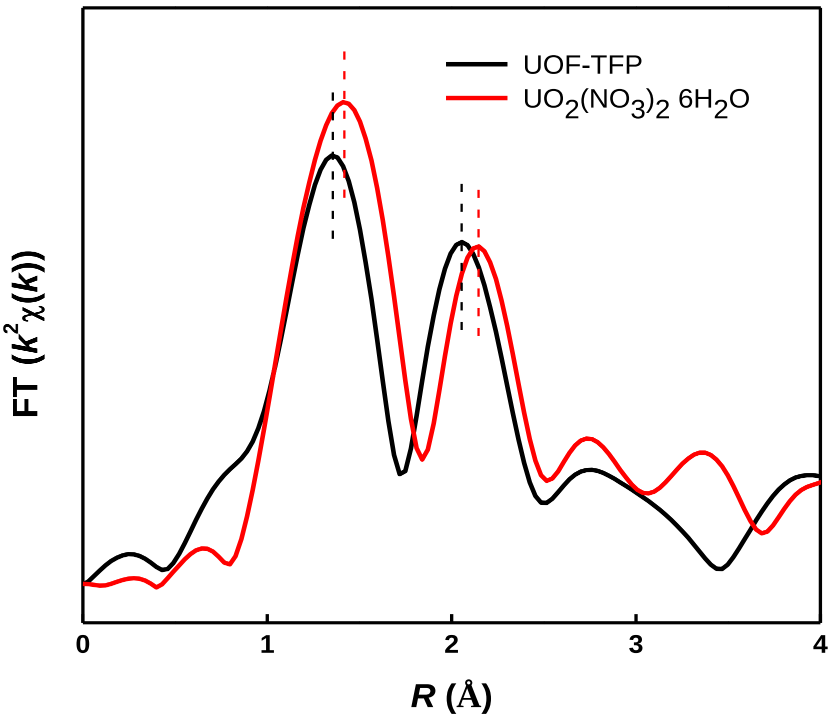

Supplementary Fig. 14| EXAFS of UOF-TFP and $UO_2(NO_3)_2 \cdot 6H_2O$.

Comment 3: The author emphasized the advantage of such UOF method. How's the cost for uranium capture from seawater as compared with some established methods?

Response: Thank you this comment. In the revision, a comparison of cost was comparied. The cost

of preparing our adsorbent of H3TFP is as low as 1.3 \$/g, which is far below the current benchmark adsorbents such as COF 4P (4.7 \$/g, *Angew. Chem. Int. Ed. 2023, 62, e202303129*) and COF-4 (2.7 \$/g, *Nat. Common. 2023, 14, 1106*)

Correction: Moreover, we should also consider the economy of uranium extraction from seawater. In generally, the manufacturing cost of adsorbents dominates the total cost for uranium extraction from seawater. For our case, the cost of preparing H_3TFP adsorbent is as low as 1.3 \$/g, confirming its economic feasibility. Such value is far below the current benchmark adsorbents such as COF 4P (4.7 \$/g)[14] and COF-4 (2.7 \$/g)[15].

(Page 9)

Comment 4: References are needed for some comparisons in the manuscript, e.g. page 9, line 213.

Response: Thank you this comment. In the revision, the references were added.

Correction: The U-O bond length of 2.40-2.70 Å is in the normal range.[33-34]

(Page 11)

Comment 5: Units should be unified, for example in Fig. 3, ppm and mg/L are mixed.

Response: Thank you this comment. In the revision, the units were unified in ppm unit (see the revised Fig. 3).

Correction:

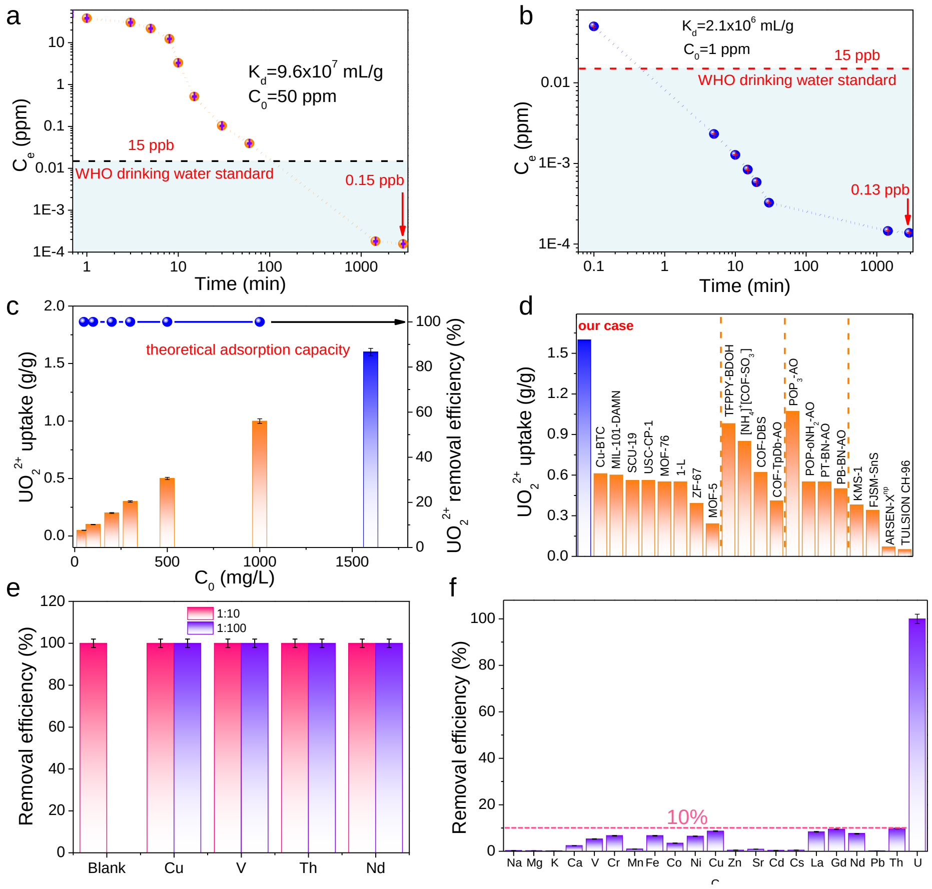

Fig. 3| Uranium adsorption upon H_3TFP. a Adsorption kinetics from 50 ppm U solution upon H_3TFP adsorbent. **b** Adsorption kinetics from 1 ppm U solution upon H_3TFP adsorbent. **c** Adsorption capacity of H_3TFP adsorbent.

d A comparison in U uptake capacity between reported U adsorbents and our case. **e** Influence of interfering ions on U adsorption. **f** Selective U capture from a 21-ions mixture solution.

Response to reviewer #2:

Thank for your valuable comments. In light of these comments, we have carefully made a revised manuscript and supplementary, which are highlighted in red in the revision.

The point-to-point response is listed as follows.

Comment 1: There is no problem using 1,3,5-triformylphloroglucinol as an adsorbent. However, there lacks the data to support the solubility of 1,3,5-triformylphloroglucinol in the water. This is very important for such MOF-based route.

Response: Thank you this comment. In the revision, the solubility of 1,3,5-triformylphloroglucinol in the water was tested. Our results strongly suggest the insolubility of 1,3,5-triformylphloroglucinol ligand in water (see Supplementary Figs. 6 and 7), thus fully meeting our demand as solid adsorbent.

Correction: In both solid- and liquid-extraction routes, a key step in the desorption process is the solid-liquid separation between $UO_2(NO_3)_2$ solution and H_3TFP precipitation regeneration from desorption. Thus, it is important to reveal the solubility of H_3TFP in water. As shown in Supplementary Fig. 6, it was clear that H_3TFP is completely soluble in ODCM, but insoluble in water. This can be further attested by UV-visible spectral test (Supplementary Fig. 7), where H_3TFP in ODCM gave a strong adsorption peak at 320 nm, while no obvious adsorption was observed for H_3TFP in water.

(Page 9)

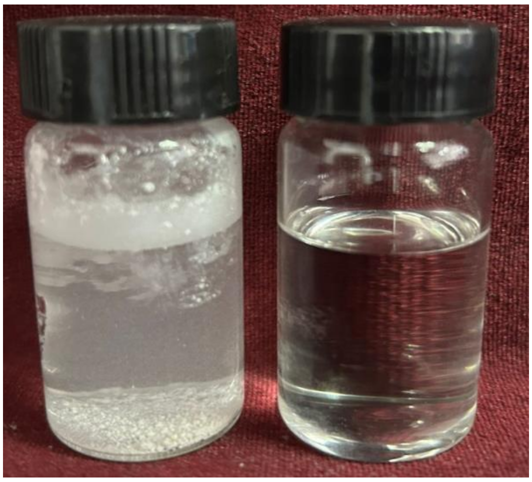

Supplementary Fig. 6| A comparison of the solution of H_3TFP in water and ODCM. Clearly, H_3TFP is insoluble in water, but completely soluble in ODCM.

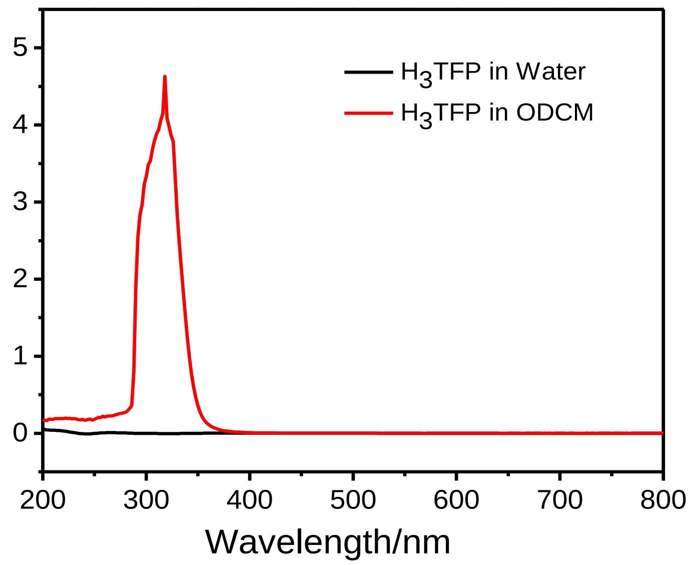

Supplementary Fig. 7| A comparison of UV-visible spectral for H_3TFP in water and ODCM. This also clearly suggests that H_3TFP is insoluble in water, but soluble in ODCM.

Comment 2: The U selectivity over Cu^{2+}, Fe^{3+}, Nd^{3+}, Th^{4+}, and VO_3^- ions are attractive. But there lack in-depth discussion in the reason. Especially for Cu^{2+} ions, it can also form d-f conjugated 2D Cu-organic framework.

Response: Thank you this comment. In the revision, we make a clear explain on the selectivity of U ion over other ions. First, the selectivity of UO_2^{2+} ion over Fe^{3+}, Nd^{3+}, Th^{4+}, and VO_3^- ions is determined by their coordination feature, *viz.* planar coordination of UO_2^{2+} ion *vs.* spherical coordination of Fe^{3+}, Nd^{3+}, Th^{4+}, and VO_3^- ions, since only planar coordination can meet the demand of formation of π-d/f conjugated 2D metal-organic framework (the uranium adsorption mechanism). Second, to disclose the selectivity of UO_2^{2+} ion over planar-coordinated Cu^{2+} ions, we further carried out DFT calculation (Supplementary Fig. 3). And we found that the binding energy between H_3TFP and UO_2^{2+} ions is far bigger than that between H_3TFP and Cu^{2+} ion, strongly suggesting higher affinity of H_3TFP towards UO_2^{2+} over Cu^{2+} ion and consequently UO_2^{2+} selectivity over Cu^{2+} ion.

Correction: The selectivity of UO_2^{2+} over Fe^{3+}, Nd^{3+}, Th^{4+}, and VO_3^- can be easy to understand, since UO_2^{2+} is planar coordinated and can effectively construct the π-f conjugated 2D uranium-organic framework, whereas other ions such as Fe^{3+}, Nd^{3+}, Th^{4+}, and VO_3^- are spherical coordinated and can not construct corresponding π-d/f conjugated 2D metal-organic framework. However, Cu^{2+} ion also own the planar coordination feature, and can also form the π-d conjugated 2D metal-organic framework, which will theoretically result in considerably competitive adsorption with UO_2^{2+} ion. Thereby, to understand the UO_2^{2+}/Cu^{2+} selectivity in this work, DFT calculation on binding energy was carried out (Supplementary Fig. 3). The negative binding energy (ΔG) of -1.16 eV implies the adsorption potential of H_3TFP ligand towards Cu^{2+} ions. But the binding towards Cu^{2+} ion is far

weaker than that towards UO_2^{2+} ion, as evidenced by the ΔG value (-1.16 eV) in Cu^{2+} ion that is far bigger than that (-3.91 eV) in UO_2^{2+} ion; thus H_3TFP ligand can enable selective UO_2^{2+} capture over Cu^{2+} ion.

(Page 5-6)

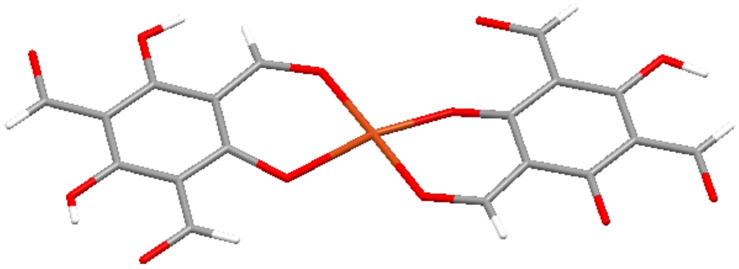

Supplementary Fig. 3| View of the optimal structure from DFT calculation for the coordination structure of Cu(II) ion with H_3TFP ligands.

Comment 3: Such precipitation method is interesting and effective. However, in light of the nature of 1,3,5-triformylphloroglucinol, can the author extend to liquid extraction by 1,3,5-triformylphloroglucinol? This will be also attractive for uranium separation, if the results are positive.

Response: Thank you this comment. In the revision, we further explored the liquid extraction by using 1,3,5-triformylphloroglucinol as liquid phase extractant. Interestingly, using 1,3,5-triformylphloroglucinol dissolved in various organic solvents as liquid phase extractant, we found that such liquid extraction from a 50 ppm U solution can be also effective (Supplementary Fig. 5 and revised Fig. 4). Especially, organic solvents of dichloromethane (DCM) and ortho-dichlorobenzene (ODCB) can lead to 100% removal within 15 min, which is equal to that observed in the solid-extraction route. Extending the contacting time up to 48 h, the uranium concentration was reduced as low as 1.24 ppb, far below the WHO standard of drinking water. These results strongly support its promising potential in liquid extraction.

Correction: Interestingly, although H_3TFP is insoluble in water, however it gave good solubility in many organic solvents such as dichloromethane (DCM), toluene (PhMe), ortho-dichlorobenzene (ODCB), p-xylene (PX), m-xylene (MX), ortho-xylene (OX), and trimethylbenzene (TB). Thus, we further developed the liquid-extraction route (Fig. 4) and the results were shown in Supplementary Fig. 5. Notably, such liquid-extraction route was found to be also effective for uranium capture. Especially, H_3TFP in DCM and ODCB was found to gave 100% removal for a 50 ppm uranium solution within 15 min. This result is comparable with that observed in the solid-extraction route. We also extended the contacting time up to 48 h for H_3TFP in DCM, and found extremely low residual concentration of uranium (1.24 ppb), which is also far below the WHO standard of drinking water.

(Page 8)

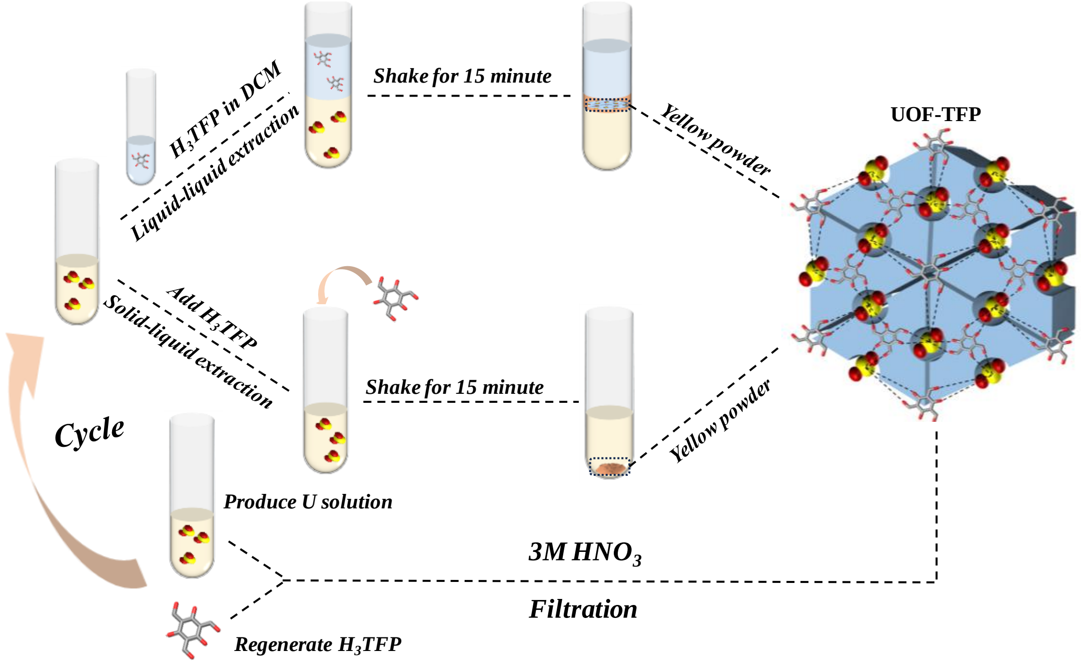

Fig. 4| Our MOF routes. View of the adsorption-desorption cycle by means of our new separation approach in both solid- and liquid-extraction routes.

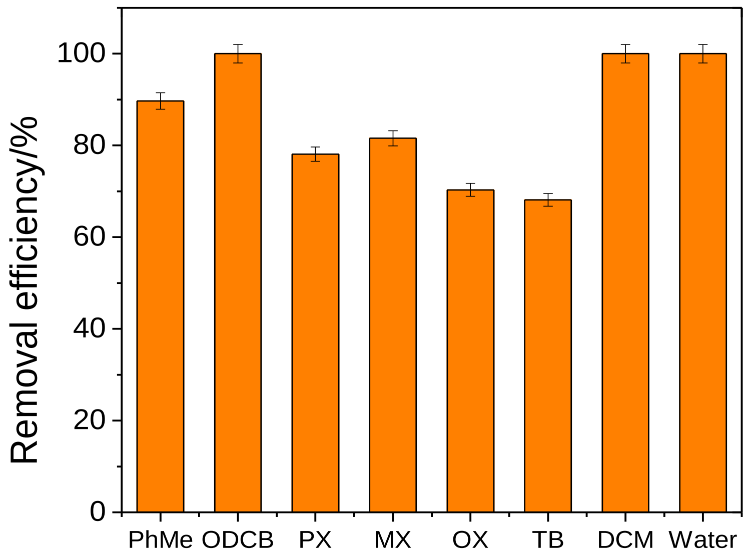

Supplementary Fig. 5| A comparison of U removal among liquid-extraction route under various organic solvents and solid-extraction route.

Comment 4: The current uranium extraction from seawater is exciting. However, I still concern about its adsorption capacity after one month, since the current adsorption capacity is till low, relative to other benchmark adsorbents.

Response: Thank you this comment. In the revision, we further explored the uranium extraction from seawater under long contacting time. Impressively, we found that extending contacting time to

10 days H_3TFP can enable as high as 6.4 mg/g uptake from 20 L natural seawater. Further extending contacting time to 14 days did not increase uptake capacity. Seen from Supplementary Table 3, the new results of 6.4 mg/g uptake for our case is clearly located on the top level in all established materials for such use.

Correction: In addition, we also conducted the uranium extraction under a longer contacting time from a 20 L natural seawater (3.3 ppb U) using 10 mg H_3TFP adsorbent. Impressively, almost 97% uranium can be captured after 10 days, giving uranium uptake as high as 6.4 mg/g, while extending contacting time up to 14 days did not increase uranium uptake. A comparison in uranium uptake between established materials and our case is shown in Supplementary Table 3, which clearly suggests our case with the location of top level in the field of uranium extraction from seawater by adsorbents.

(Page 9)

Comment 5: The title is long. 'Ultra-selective uranium separation by in-situ formation of π-f conjugated 2D uranium-organic framework' is more proper.

Response: In the revision, the title was corrected as 'Ultra-selective uranium separation by in-situ formation of π-f conjugated 2D uranium-organic framework'.

Comment 6: Page 1, line 9, 'has' should be 'have'. Please also check other grammar and spelling errors.

Response: In the revision, we carefully checked the manuscript, and all these grammar and spelling errors were completely corrected.

Comment 7: Page 6, there lacks a discussion to explain the pH effect.

Response: Thanks for this comment. In the revision, a discussion of pH effect was added.

Correction: High acidity leading to a sharp decrease in uranium uptake is mainly due to the protonation that will significantly affect the coordination of H_3TFP hydroxyl groups, and high alkalinity resulting in a sharp decrease in uranium uptake is mainly due to the solution of H_3TFP under such condition that will significantly affect the UOF formation surrounding.

(Page 7)

Comment 8: Page 9, IR of $UO_2(NO_3)_2\cdot 6H_2O$ is suggested to be tested.

Response: Thank you this comment. In the revision, we further measured the IR of $UO_2(NO_3)_2\cdot 6H_2O$ (Supplementary Fig. 8).

Correction:

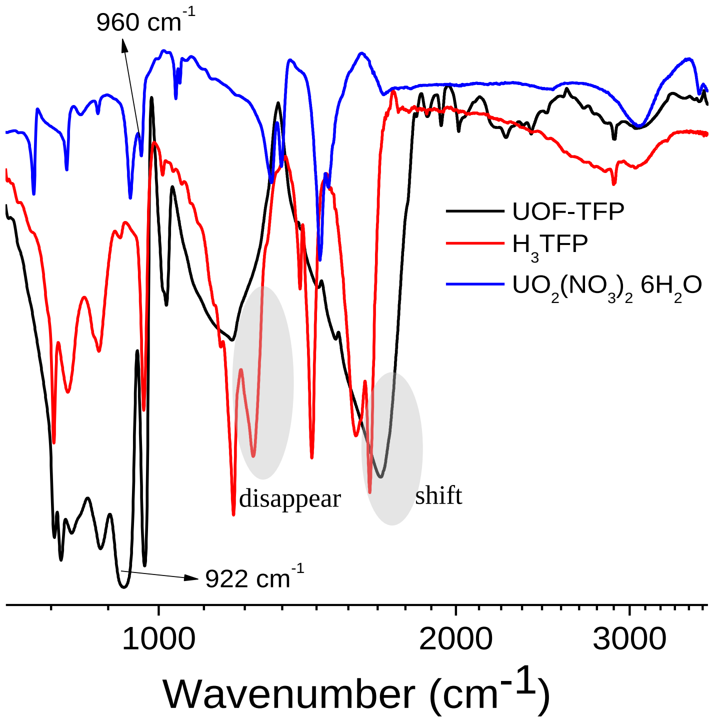

Supplementary Fig. 8| IR spectra of UOF-TFP, H_3TFP ligand, and $UO_2(NO_3)_2\cdot6H_2O$.

Response to reviewer #3:

Thank for your helpful comments. In light of these comments, we have carefully made a revised manuscript and supplementary, which are highlighted in red in the revision.

The point-to-point response is listed as follows.

Comment 1: why is H_3TFP ligand the best? what is relationship between uranium capture and organic ligand? who is the key factor?

Response: Thank you this comment. To make clear the above questions, in the revision we further made DFT calculation on these ligands. The biggest binding energy between UO_2^{2+} and H_3TFP explained the best uranium capture performance in H_3TFP. The negative binding energy for all these ligands strongly suggests the adsorption thermodynamically spontaneous in a mode of formation of π-f conjugated 2D uranium-organic framework. A comparison in binding energy, the coordination structure and coplanarity, it was found that the chelate coordination in the combination of one aldehyde oxygen and one hydroxyl oxygen is more beneficial for U-O coordination and planar coordination configuration, which could be the key to determine the UO_2^{2+} uptake performance. In addition, big conjugated structure is also more beneficial for planar coordination configuration and consequently UO_2^{2+} uptake. And a discussion was added in the revision (see page 3-4, highlight in red, Supplementary Fig. 1).

Correction: To clarify the difference in UO_2^{2+} capture for these organic ligands, we then carried out the calculation on the binding energy (ΔG) of these organic ligands with UO_2^{2+} ion by density functional theory (DFT) method. A planar six-coordination model of one UO_2^{2+} ion coordinated by three these organic ligands was used to carried out DFT calculation. The optimized coordination structures of them were shown in Supplementary Fig. 1. The binding energy (ΔG) gives a hierarchy of H_4THQ (-0.29 eV)>H_6HTP (-0.69 eV)>H_4EAA (-1.02 eV)>H_2HPD (-2.99 eV)>H_3TFP (-3.91 eV). Generally, negative binding energy (ΔG) suggests the reaction thermodynamically spontaneous, while this also obeys a rule, *viz.* the more negative, and the stronger the binding. Thus, the negative ΔG values mean that all these organic ligands can capture UO_2^{2+} ion through coordination, which is consistent with the experimental results, while the smallest and biggest ΔG value in respectively H_3TFP and H_4THQ means the strongest and weakest binding and consequently the biggest and smallest UO_2^{2+} uptake, which is also in good agreement with the experimental results. Moreover, the hierarchy in binding energy (ΔG) is also in accord with the hierarchy in the UO_2^{2+} uptake, confirming the adsorption of UO_2^{2+} ions by these organic ligand obeying the defined planar coordination principle. In addition, seen from the optimized coordination structures of them, it is found that the chelate coordination in the combination of one aldehyde oxygen and one hydroxyl oxygen is more beneficial for U-O coordination and planar coordination configuration such as H_4TFP and H_2HPD over the chelate coordination from two hydroxyl oxygens such as H_4EAA, H_6HTP, H_4THQ, due to smaller steric hindrance in the combination of one aldehyde oxygen and one hydroxyl oxygen. And this could be the key to determine the UO_2^{2+} uptake performance. Furthermore, big conjugated structure is also more beneficial for planar coordination configuration and consequently UO_2^{2+} uptake, e.g. H_6HTP and H_4EAA vs. H_4THQ.

(Page 3-4)

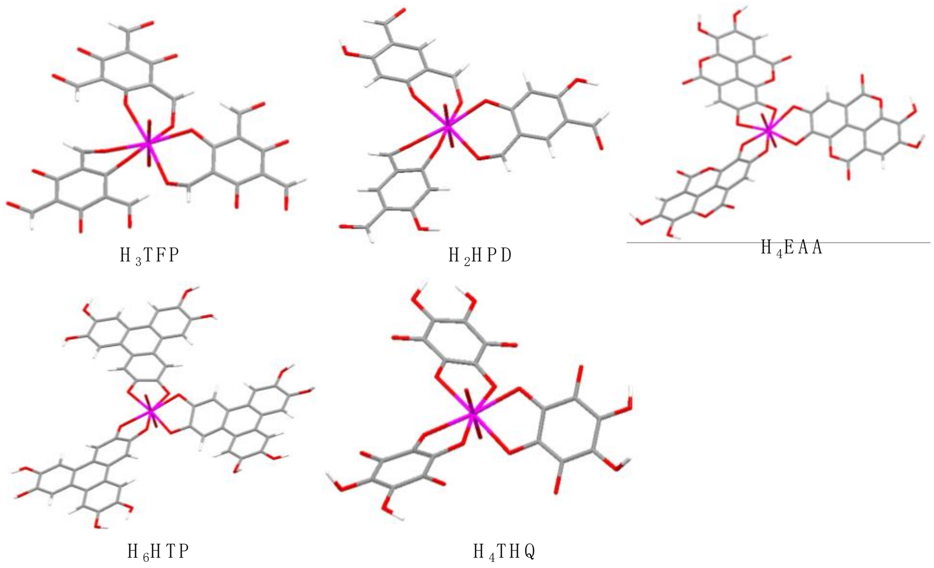

Supplementary Fig. 1| View of the coordination structures between UO_2^{2+} ions and these organic ligands.

Comment 2: why is the uranium selectivity better over other ions using H3TFP ligand?

Response: Thank you this comment. To make clear the above question, in the revision we further made an in-deepth discussion and DFT calculation. First, the selectivity of UO_2^{2+} ion over Fe^{3+}, Nd^{3+}, Th^{4+}, and VO_3^- ions is determined by their coordination feature, *viz.* planar coordination of

UO_2^{2+} ion *vs.* spherical coordination of Fe^{3+}, Nd^{3+}, Th^{4+}, and VO_3^- ions, since only planar coordination can meet the demand of formation of π-d/f conjugated 2D metal-organic framework (the uranium adsorption mechanism). Second, to disclose the selectivity of UO_2^{2+} ion over planar-coordinated Cu^{2+} ions, we further carried out DFT calculation (Supplementary Fig. 3). And we found that the binding energy between H_3TFP and UO_2^{2+} ions is far bigger than that between H_3TFP and Cu^{2+} ion, strongly suggesting higher affinity of H_3TFP towards UO_2^{2+} over Cu^{2+} ion and consequently UO_2^{2+} selectivity over Cu^{2+} ion.

Correction: The selectivity of UO_2^{2+} over Fe^{3+}, Nd^{3+}, Th^{4+}, and VO_3^- can be easy to understand, since UO_2^{2+} is planar coordinated and can effectively construct the π-f conjugated 2D uranium-organic framework, whereas other ions such as Fe^{3+}, Nd^{3+}, Th^{4+}, and VO_3^- are spherical coordinated and can not construct corresponding π-d/f conjugated 2D metal-organic framework. However, Cu^{2+} ion also own the planar coordination feature, and can also form the π-d conjugated 2D metal-organic framework, which will theoretically result in considerably competitive adsorption with UO_2^{2+} ion. Thereby, to understand the UO_2^{2+}/Cu^{2+} selectivity in this work, DFT calculation on binding energy was carried out (Supplementary Fig. 3). The negative binding energy (ΔG) of -1.16 eV implies the adsorption potential of H_3TFP ligand towards Cu^{2+} ions. But the binding towards Cu^{2+} ion is far weaker than that towards UO_2^{2+} ion, as evidenced by the ΔG value (-1.16 eV) in Cu^{2+} ion that is far bigger than that (-3.91 eV) in UO_2^{2+} ion; thus H_3TFP ligand can enable selective UO_2^{2+} capture over Cu^{2+} ion.

(Page 5-6)

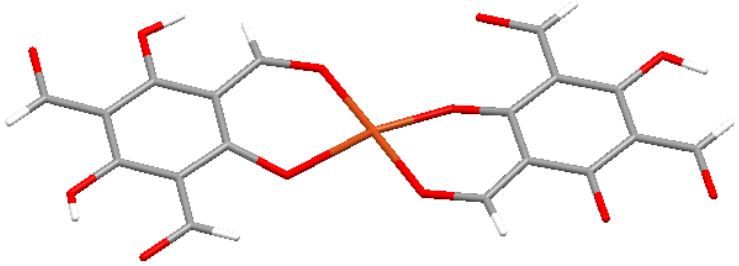

Supplementary Fig. 3| View of the optimal structure from DFT calculation for the coordination structure of Cu(II) ion with H_3TFP ligands.

Comment 3: Assuming such precipitation method with this mechanism of in-situ formation of π-f conjugated 2D UOF, however, we are not clear about the coordination number and bond length, which calls additional characterizations in this aspect.

Response: Thank you this comment. To make clear the above question, we further measured a EXZAF spectra (see Supplementary Figs. 13, 14, Table 4, and Fig. 6). And we can directly read out the coordination number and bond length through fitting EXZAF data, giving eight coordination with three types of bond length; this result is in good agreement with the simulated structure.

Correction: In addition, to gain deep insight into the local coordination sphere of the uranium species, U K-edge X-ray absorption near-edge structure (XANES, Supplementary Fig. 13) and extended X-ray absorption

fine structure (EXAFS, Supplementary Fig. 14) spectroscopy were performed. $UO_2(NO_3)_2 \cdot 6H_2O$ was used as the reference standard. It was found that UOF-TFP rendered comparable XANES and EXAFS spectra with that observed in $UO_2(NO_3)_2 \cdot 6H_2O$, confirming their similarity in the valence state and coordination surrounding of uranium. As we know, uranium in $UO_2(NO_3)_2 \cdot 6H_2O$ is hexavalent in the form of UO_2^{2+}, and UO_2^{2+} in $UO_2(NO_3)_2 \cdot 6H_2O$ takes planar six coordination with two NO_3^- ions and two coordination water molecules, where each NO_3^- ion displays a chelate coordination mode with two NO_3^- oxygen atoms to fix one UO_2^{2+} ion. Accordingly, we can deduce also UO_2^{2+} ions with also the planar six coordination in UOF-TFP (Supplementary Table 4). High-quality fits of the EXAFS data for UOF-TFP (Figs. 6a and 6b) strongly suggest an eight coordination of uranium, where two U-O coordinations with bond length of 1.85±0.01 Å are assigned to U=O bond of UO_2^{2+} ion, other six U-O coordinations with longer bond length are assigned to the planar U-O coordination from TFP^{3-} ligand. This also reveals two types of U-O bond with equal numbers in the bond length of 2.42±0.02 Å and 2.51±0.01 Å, respectively. We then comparied the results from EXAFS data with the results from structural simulation, and found that the U=O bond of UO_2^{2+} ion form EXAFS data is well consistent with the results from structural simulation (1.84 Å), while the bond length of 2.42±0.02 Å and 2.51±0.01 Å from EXAFS data is close to $U\text{-}O_{hydroxyl}$ bond (2.40 Å, hydroxyl oxygen of TFP^{3-} as coordination atom) and $U\text{-}O_{aldehyde}$ bond (2.70 Å, aldehyde oxygen of TFP^{3-} as coordination atom) from structural simulation. Thereby, the structure of UOF-TFP can be once again confirmed by EXAFS results.

(Page 12)

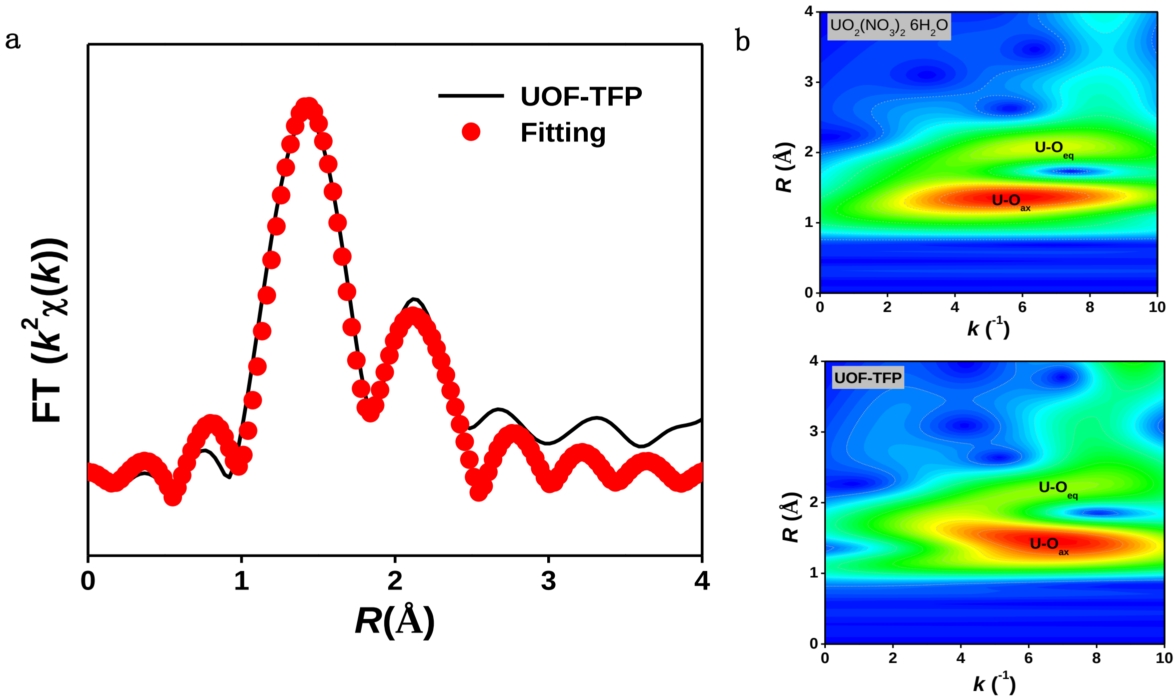

Fig. 6| EXZAF of UOF-TFP. a Experimental EXZAF data (red) and fitting results (black). **b** View of coordination surrounding of UOF-TFP and $UO_2(NO_3)_2 \cdot 6H_2O$, where $U\text{-}O_{ax}$ represents the U=O coordination bonds, $U\text{-}O_{eq}$ represents the $U\text{-}O_{nitrate}$ *plus* $U\text{-}O_{water}$ in $UO_2(NO_3)_2 \cdot 6H_2O$ and $U\text{-}O_{hydroxyl}$ *plus* $U\text{-}O_{aldehyde}$ in UOF-TFP.

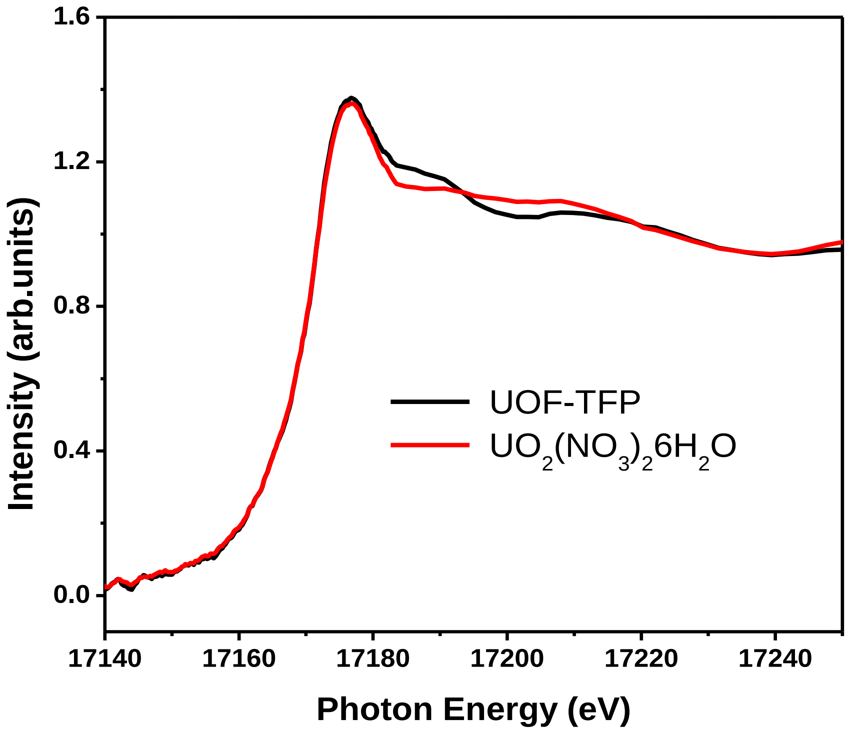

Supplementary Fig. 13| XANES of UOF-TFP and $UO_2(NO_3)_2 \cdot 6H_2O$.

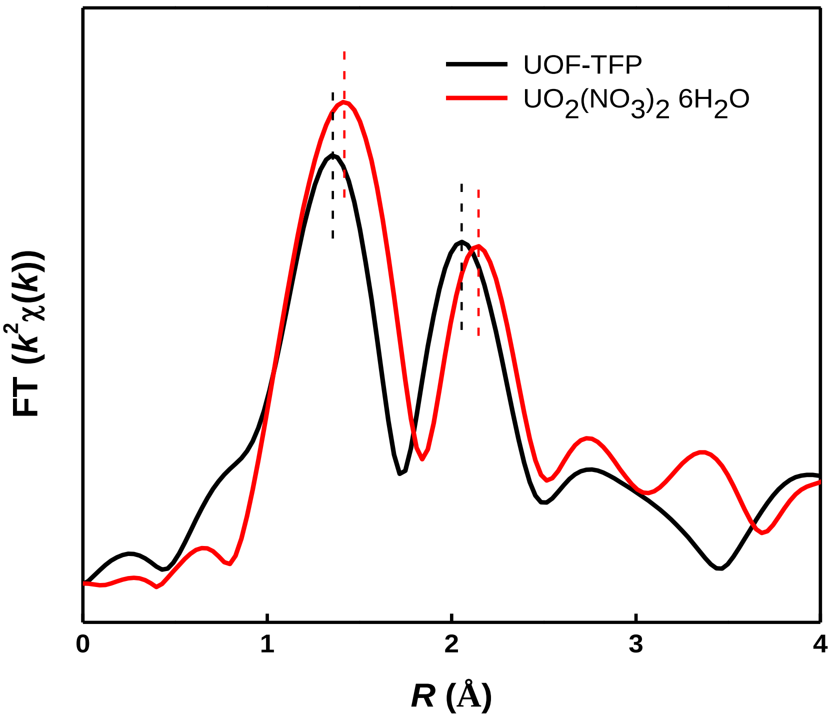

Supplementary Fig. 14| EXAFS of UOF-TFP and $UO_2(NO_3)_2 \cdot 6H_2O$.

REVIEWERS' COMMENTS

Reviewer #1 (Remarks to the Author):

The authors have satisfactorily addressed all the comments from the reviewer and the manuscript can now be accepted in its current form.

Reviewer #2 (Remarks to the Author):

I believe authors have fully addressed my comments during revisions.

Reviewer #3 (Remarks to the Author):

The authors have successfully addressed my comments and the manuscript is now recommended to be accepted by the journal.
